# Circuit logic of oxytocin and vasopressin complementary actions

**DOI:** 10.3389/fnins.2026.1875731

**Published:** 2026-07-17

**Authors:** Freddy Jeanneteau, Prabahan Chakraborty, Hugues Petitjean, Alexandre Charlet

**Affiliations:** 1 Institut de Génomique Fonctionnelle, University Montpellier, CNRS, Inserm, Montpellier, France; 2 Department of Genetic Engineering, SRM Institute of Science and Technology, Kattankulathur, Tamil Nadu, India; 3 Institute of Cellular and Integrative Neuroscience, Centre National de la Recherche Scientifique and University of Strasbourg, Strasbourg, France

**Keywords:** circuit motif, neuromodulation, psychiatry, push-pull, social

## Abstract

Oxytocin (OT) and vasopressin (VP) are evolutionarily conserved neuropeptides that regulate social behavior, emotional processing and physiological homeostasis. Although traditionally studied as individual modulators of affiliation, stress and autonomic function, emerging evidence indicates that their actions are best understood at the level of neural circuits. Advances in optogenetics, cell-type-specific electrophysiology and systems neuroscience have revealed that OT and VP act within distributed networks through receptor-defined microcircuits composed of excitatory and inhibitory neuronal populations, astrocytes and long-range projections. Within these circuits, OT and VP can exert complementary, synergistic or opposing effects depending on receptor localization, cellular identity and network state. Here, we synthesize recent circuit-level and electrophysiological evidence to propose a framework in which OT and VP operate as a coordinated neuromodulatory axis. We argue that the functional consequences of OT/VP signaling emerge not from peptide identity alone, but from their engagement of recurrent circuit motifs that redistribute excitation and inhibition across neural networks. These motifs provide a mechanistic substrate for regulating transitions between competing behavioral and physiological states, including social safety vs. threat, affiliation vs. avoidance, and parasympathetic vs. sympathetic dominance. We further discuss how disruption of receptor topology, synaptic integration and circuit architecture may contribute to neurodevelopmental, psychiatric and stress-related disorders. By shifting the focus from peptide-centric models to circuit-level mechanisms, this framework reconciles seemingly contradictory findings across brain regions and behavioral paradigms, and provides a foundation for the development of next-generation circuit-based therapeutic strategies targeting the oxytocin-vasopressin axis.

## 1 Introduction

Oxytocin (OT) and vasopressin (VP) were conceptualized as hypothalamic-derived hormones exerting broad modulatory actions through diffuse volume transmission in brain and body. While such mechanisms undoubtedly exist, OT and VP also operate through precise, temporally controlled, and spatially restricted release within defined neural circuits (Stoop, 2012).

This view is compatible with recent brain circuit-level evidence demonstrating spatially precise release, synapse-specific receptor distribution, and fast electrophysiological effects in genetically defined neuronal populations (Knobloch et al., 2012; Neumann and Landgraf, 2012; Eliava et al., 2016; Grinevich et al., 2016; Hasan et al., 2019; Tang et al., 2020; Iwasaki et al., 2023; Bortolozzo-Gleich et al., 2025; François et al., 2025). Rather than exerting uniform effects, OT and VP shape neural computations in a context-dependent and circuit-specific manner (Grinevich and Stoop, 2018). Hence, OT and VP should not be viewed as unitary “prosocial vs. antisocial” or “parasympathetic vs. sympathetic” systems. Instead, they constitute interacting control signals embedded in recurrent macro- and micro-circuits. Notably, their actions are often *concerted*, reinforcing similar network outputs, and at times *oppositional*, producing antagonistic behavioral states (Raggenbass, 2008).

**Figure 1:**
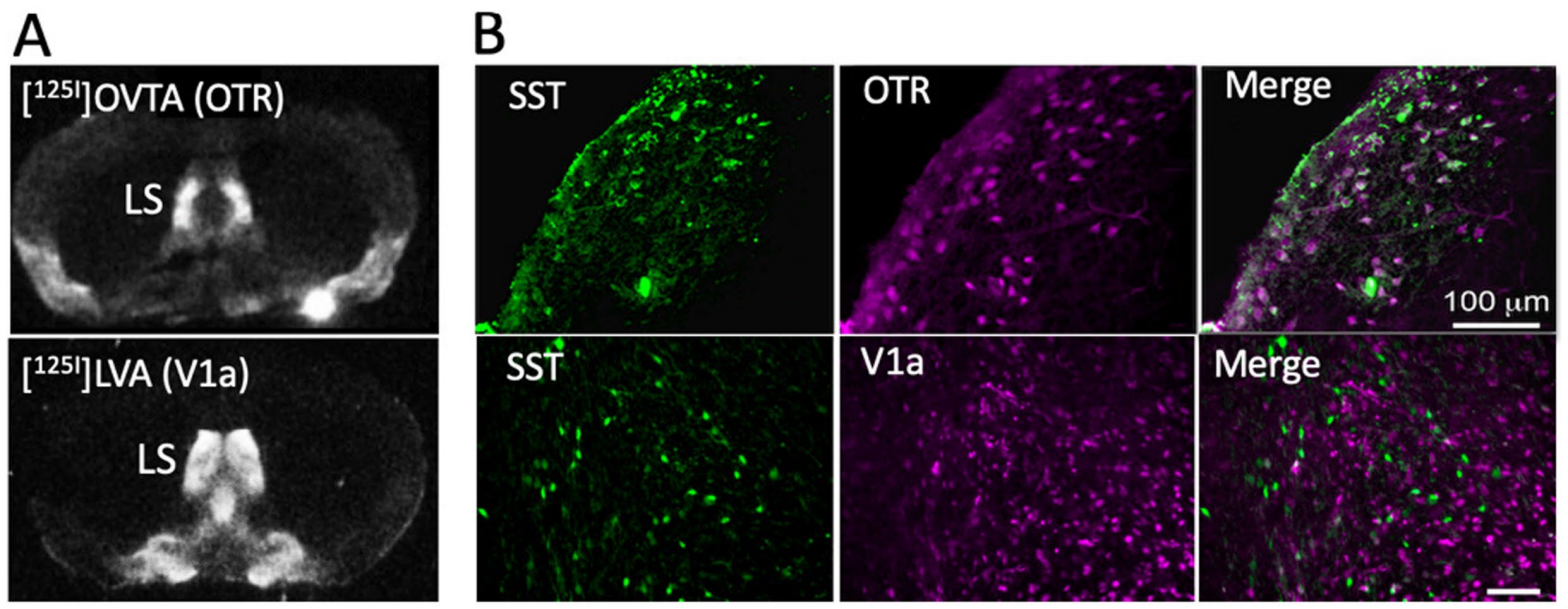
Fluorescent tracers reveal juxtaposed and segregated expression of OTR and V1a in the brain. **(A)** Radiotracers are highly specific but lack cellular precision in the mouse brain. **(B)** Fluorescent peptide d[Lys(Alexa Fluor 647)8]VP marks OTR-expressing cells in the presence of Manning compound (top panels adapted from Borie et al. 2024), or V1a-expressing cells in the presence of TGOT (bottom panels). In the mouse lateral septum, OTR is very abundant in SST neurons unlike V1a segregated in neighboring cells.

To resolve this paradox, critically examining the microcircuit architecture composed of excitatory and inhibitory neurons in which these peptides operate provides answers. The emergent behavioral output is not determined by peptide identity alone, but by the topological embedding of their receptors within synaptic loops. New fluorescent ligands (Corbani et al., 2018; Borie et al., 2021a, 2024; Marciniak et al., 2022; Nakamura et al., 2022) for visualizing in real time the receptors in living systems have improved detection with cellular precision (Figure 1) beyond the technical limitations of classical radiotracers (macroresolution) and genetic tags (permanent labeling inconsistent with receptor levels).

Across brain regions, OT and VP receptors are frequently distributed in adjacent or complementary neuronal populations, forming the anatomical basis for *coordinated* yet *opposing* modulation (Song and Albers, 2018; Smith et al., 2019). Within such circuits, OT often enhances inhibitory control or promotes network stability (Tirko et al., 2018; Zhang et al., 2024), whereas VP tends to enhance excitation or output signaling (Huber et al., 2005; Borie et al., 2021a; François et al., 2025). These local interactions can scale up to influence complex behaviors, including social recognition (Borie et al., 2024), nociception (Juif and Poisbeau, 2013), fear responses (Huber et al., 2005), and autonomic regulation (Viviani et al., 2011).

In this review, we integrate molecular, cellular, and systems-level findings to propose a unifying conceptual framework from *peptide-to-behavior mapping* toward *peptide-to-microcircuit transformation*, where behavioral outcomes emerge from the interaction between peptidergic signaling and circuit topology. This framework is especially evident in limbic structures such as the amygdala and lateral septum, where OT and VP act on adjacent but non-overlapping neuronal ensembles that form excitatory-inhibitory gating motifs. This logic can be extended to other motifs found in, but not limited to, the bed nucleus of *Stria Terminalis*, olfactory bulb, hippocampus, brainstem and spinal cord. We examine this framework across key behavioral domains and discuss its relevance for neurodevelopment and psychiatric manifestations.

## 2 Principles

The diverse functions of OT and VP can be interpreted through a set of organizational principles that transcend individual brain regions. Although these principles emerge from observations in specific circuits, we propose that they provide a useful framework for understanding how OT and VP shape neural computations across distributed networks.

A first principle is the spatial segregation of OT and VP receptor expression. OTR, V1a and V1b receptors are frequently expressed in adjacent, but distinct, neuronal populations, creating an anatomical substrate for complementary or opposing modulation. In several limbic structures, OTR expression is enriched in inhibitory interneurons, whereas V1a receptors are preferentially associated with excitatory populations. As a result, OT often influences circuit output indirectly through inhibition or disinhibition, whereas VP more commonly enhances network activity through direct excitation or presynaptic regulation of neurotransmitter release (Leroy et al., 2018; Borie et al., 2022). Importantly, these patterns are not universal and vary across regions, cell types and physiological states. Nevertheless, receptor segregation provides a structural basis through which OT and VP can either converge on a common output or bias activity toward competing network states. Because receptor expression is dynamically regulated by developmental stage, sex and experience, these variables represent critical determinants of OT/VP function (Smith et al., 2017; François et al., 2025).

A second principle is signaling flexibility at the receptor level. OTR, V1a and V1b receptors engage multiple intracellular pathways whose recruitment varies according to cell type, physiological state and ligand concentration. OTR can couple to G^q/11-^, G^i/o-^ and *β*-arrestin-dependent signaling cascades, whereas V1a and V1b primarily signal through G^q^ pathways and V2 receptors through Gs-mediated mechanisms (Schöneberg et al., 1998; Busnelli et al., 2012; Koshimizu et al., 2012; Baudon et al., 2025). Synthetic ligands like Atosiban and DNalOVT proved that exclusive recruitment, by OTR, of G_i_ (but not G_q/11_) or β-Arrestin uncoupling can generate specific physiological and behavioral consequences. On the other hand, carbetocin provides a strongly biased recruitment of the G_q_ pathway (Busnelli et al., 2012; Busnelli and Chini, 2017). This signaling diversity enables a single peptide to produce distinct, and occasionally opposing [via KCC2 GABA switch by OTR (Leonzino et al., 2016) or V2 (Welker et al., 2008)], effects on neuronal excitability (Gravati et al., 2010), synaptic transmission and cellular plasticity, astrocyte morphology and gliotransmission (Wahis et al., 2021; Baudon et al., 2025). Functional outcomes are further complicated by receptor cross-reactivity (Sala et al., 2011; Inada et al., 2025), as OT and VP can activate each other’s receptors under specific conditions (Bichet et al., 2019; Elsaafien et al., 2026). Consequently, the effects of peptide release depend not only on receptor identity but also on receptor density, coupling state, peptide concentration and release dynamics (Landgraf and Neumann, 2004). Recent optogenetic, chemogenetic and photopharmacological studies further indicate that firing patterns of OT and VP neurons govern both the spatial extent and temporal profile of peptide signaling, generating responses that range from rapid synaptic modulation to prolonged network reconfiguration (Knobloch et al., 2012; Eliava et al., 2016; Grund et al., 2019; Iwasaki et al., 2023; Dromard et al., 2024; Rigney et al., 2024; Bortolozzo-Gleich et al., 2025; Francesconi et al., 2025; François et al., 2025).

A third principle is that the consequences of OT and VP signaling are determined as much by circuit topology as by receptor pharmacology. Identical receptor-mediated effects at the cellular level can generate opposite behavioral outcomes depending on whether they occur within excitatory or inhibitory nodes of a network (Stoop, 2012). Thus, apparent functional opposition between OT and VP often emerges from their differential embedding within local microcircuits rather than from intrinsically antagonistic receptor actions. We discuss three recurrent motifs through which these effects are implemented: push–pull modulation, synergistic amplification and parallel pathway segregation (Table 1).

**Table 1:** Key terms.

Circuit motif	Definition
Synergistic Amplification	Increases circuit gain through complementary of VP-driven excitation and OT-mediated inhibition at distinct nodes within a circuit motif.
Push-Pull modulation	Enhances discrimination through complementary actions of VP and OT: VP increases excitatory drive in upstream neurons, whereas OT suppresses downstream output via recruitment of inhibitory pathways. This opposition filters salient signals from background noise across a circuit motif.
Parallel Pathways Segregation	Occurs when VP and OT modulate distinct, non-converging pathways that nonetheless coordinate a common behavioral output.

A fourth principle concerns temporal organization. Unlike classical neurotransmitters, OT and VP are preferentially released during sustained or high-frequency neuronal activity, and their effects often evolve over timescales ranging from seconds to hours (Rossoni et al., 2008; van den Pol, 2012; Brown et al., 2013). These dynamics allow OT and VP to coordinate behavioral states across extended temporal windows rather than mediate moment-to-moment synaptic transmission alone (Borie et al., 2021a; Zhang et al., 2024). The duration and magnitude of signaling are further shaped by receptor trafficking, desensitization and heterodimerization, adding layers of regulation to an already highly context-dependent system (Jurek and Neumann, 2018).

Together, these observations support a shift from a peptide-centric to a circuit-centric view of OT/VP signaling. Although OT/VP-responsive circuits are not organized according to a universal blueprint, the recurrence of similar microcircuit motifs across brain systems suggests a common operational logic. In this framework, behavioral outcomes emerge primarily from circuit topology, receptor distribution and network state, with peptide identity acting as only one component of a broader computational architecture (Table 2).

**Table 2:** Summary.

Brain region	Receptor distribution	OT effect	VP effect	Circuit motif	Behavioral outcomes	Reference
Rat central amygdala (CeA)	OTR on GABA neurons in CeL, V1a on excitatory neurons in CeM	OT from PVN inhibit GABA neurons synapsing onto V1a neurons	VP from PVN excites excitatory neurons that OTR inhibitory neurons seek to suppress	Push-Pull modulation	Reduced freezing, arousal and anxiety	Huber et al. (2005)
Mouse olfactory bulb (OB)	V1a on mitral cells (MC) in main olfactory bulb, OTR on projecting excitatory neurons of anterior olfactory nucleus that indirectly feedback to MC via granule cells as relay	OT from PVN facilitates excitation of excitatory neurons	VP from PVN or OB excites mitral cells	Synergistic amplification and feedback inhibition via granule cells	Olfaction and social discrimination	Tobin et al. (2010); Osako et al. (2001); Dluzen et al. (1998); Ferguson et al. (2000)
Mouse bed nucleus of stria terminalis (BNST)	OTR on CRF/PKC∆, V1a not characterized	OT from PVN excites CRF/OTR/PKC∆ neurons in BNST	VP from BSNT, PVN, SCN and SON excites presumed pyramids in BNST	Synergistic amplification	Extinction of anxious arousal	Francesconi et al. (2025)
Rat lateral septum (dorsal) or hippocampus (dorsal or ventral)	Receptors not determined	OT anti-serum (antagonist) locally injected in vHipp blocks social recognition but not when injected in dHipp or dLS	VP anti-serum (antagonist) in icv., or locally injected in vHipp, dHipp or dLS block Social recognition	Synergistic amplification	Social discrimination with juveniles	Van Wimersma Greidanus and Maigret (1996)
Mouse lateral septum (dorsal)	V1a on CRF2/NDNF neurons, OTR on SST neurons	OT from PVN (via GABA_B_) inhibits LS SST neurons and briefly increased	VP from BNST excites CRF2/NDNF neurons that inhibit LS SST neurons (via GABA_A_) and stabilized increased theta oscillations	Synergistic amplification	Social discrimination with juveniles	Borie et al. (2021a)
Mouse lateral septum (dorsal)	V1a on CRF2/NDNF neurons, OTR on SST neurons	OT from PVN (via GABA_B_) inhibits LS SST neurons	VP from PVN excites CRF2/NDNF neurons that inhibit LS SST neurons (via GABA_A_)	Synergistic amplification	Social novelty, familiarization and discrimination with juveniles	Borie et al. (2024)
Mouse lateral septum (dorsal)	V1a on CRF2/NDNF neurons, OTR on SST neurons	OT from SON (via GABA_B_) inhibits LS SST neurons	VP from SON excites CRF2/NDNF neurons that inhibit LS SST neurons (via GABA_A_)	Synergistic amplification	Social fear extinction, aggression with adults	Dromard et al. (2024)
Rat lateral septum	V1a in dorsal LS GABA neurons, OTR GABA neurons in ventral LS	OT from PVN and SON decreases aggression of single housed females, opposite if group-housed	VP from PVN and SON decreases aggression of single house females, opposite if group-housed	Synergistic amplification	Lactating female aggression	Oliveira et al. (2021)
Rat hippocampus	Receptors not determined	OT injection attenuates theta oscillations and passive avoidance	VP injection facilitates passive avoidance	Opposing effects	Passive avoidance	Bohus et al. (1978)
Rat hippocampus (CA1)	Receptors not determined	OT excites CA1 pyramids and interneurons	VP excites CA1 pyramids and interneurons	Synergistic amplification	Not determined	Mühlethaler et al. (1984)
Mouse hippocampus (CA2)	V1b on CA2 pyramids (axons to lateral septum), OTR on CA2 pyramids (somatodendritic)	OT from PVN inhibits excitatory postsynaptic events in CA2 pyramids	VP from PVN facilitates excitatory postsynaptic events in CA2 pyramids	Opposing effects	Social memory	Cymerblit-Sabba et al. (2023)
Mouse brain	Brain-wide	OT from intranasal administration mostly activates composite fMRI map	VP from intranasal administration mostly deactivates composite fMRI map	Opposing effects	Not determined	Galbusera et al. (2017)
Rat hippocampus Lateral septum	OTR, V1a, V1b in hippocampus and lateral septum	OT increases short-term activity in the theta band	VP increases long lasting activity in the theta band	Complementary effects	Not determined	Urban (1981); Urban (1998)
Mouse hypoglossal nucleus	OTR in glycinergic neurons projecting to V1a-expressing excitatory neurons synapsing onto preganglionic motorneurons	OT increases inhibitory postsynaptic events but the opposite at birth via KCC2	VP increases excitatory postsynaptic events	Opposing effects	Motor functions like sucking, feeding, chewing, breathing	Wrobel et al. (2010)
Human and mouse spinal cord	OTR in glycinergic neurons and excitatory neurons of the dorsal spinal cord, V1a in motorneurons of the ventral horn	OT facilitates transmission at excitatory neurons projecting to NK1 cells (noxious touch), and also at glycinergic neurons projecting to GPR83 cells (pleasurable touch)	VP increases excitatory postsynaptic events in motor neurons receiving inhibitory tone by OTR-expressing glycinergic neurons (presumed)	Parallel pathways	Social touch, pain sensitivity, motor functions	Bohic et al. (2026); Peng et al. (2015)
Mouse pre-optic area	OTR in preoptic area (cells not defined), V1a (not defined)	OT from PVN promotes paternal care.	VP from PVN promotes paternal care. Optogenetic activation of VP neurons in PVN failed to activate OT neurons in PVN	Receptor crosstalk	Paternal care and avoid pup-directed aggression	Inada et al. (2025)
Mouse spinal cord	OTR in dorsal horn spinal cord, V1a in ventral horn motorneurons and in DRG neurons	OT promotes analgesia in OTR knockouts but not in V1a knockouts	VP increases analgesia in OTR knockouts but not in V1a knosckouts	Receptor crosstalk	Analgesia	Schorscher-Petcu et al. (2010)

## 3 Processing emotions: modulation in amygdala and BNST

### 3.1 Circuit architecture

A particularly compelling illustration of the coordinated yet opposing roles of OT and VP emerges within the central amygdala (CeA) and extended amygdala circuitry. This network, which includes the bed nucleus of the *stria terminalis* (BNST), functions as a central integrator of emotionally salient information and orchestrates behavioral and autonomic responses to threat. The CeA, in particular, is uniquely positioned at the interface between sensory processing and motor output, receiving convergent inputs from cortical and subcortical structures and projecting to hypothalamic and brainstem nuclei that mediate defensive behaviors. At the core of CeA function lies a highly structured microcircuit architecture composed of GABAergic neurons organized into functionally distinct subnuclei. The lateral subdivision (CeL) acts as an input and processing hub, whereas the medial subdivision (CeM) serves as the principal output node. This internal organization provides a substrate for neuromodulatory systems to exert fine control over the flow of information from sensory inputs to behavioral outputs. Within this framework, OT and VP engage *complementary* yet *antagonistic* mechanisms that dynamically regulate the balance between inhibition and excitation across these subcircuits (Huber et al., 2005; Stoop et al., 2015).

### 3.2 Circuit function

OT exerts its primary influence within the CeA subcircuit by targeting OTR-expressing neurons and astrocytes in the CeL (Huber et al., 2005; Knobloch et al., 2012; Wahis et al., 2021). CeL GABAergic interneurons project to the CeM and form a potent inhibitory pathway that gates the activity of output neurons. Recruitment of OTR within the CeL leads to increased synaptic strength and neuronal firing, resulting in enhanced inhibitory transmission onto CeM neurons (Viviani et al., 2011; Baudon et al., 2025). This disynaptic mechanism effectively suppresses the output of the CeA, attenuating downstream activation of hypothalamic and brainstem centers involved in fear expression (Viviani et al., 2011), reducing autonomic arousal, freezing behavior, and anxiety (Stoop, 2012).

In contrast, VP acts predominantly within the CeM through activation of V1a expressed on projection neurons (Huber et al., 2005). This direct excitation, increasing their firing rate and enhancing output to downstream effectors, promotes the expression of fear-related behaviors (e.g., bradycardia, vigilance, and defensive responses; Stoop, 2012). A small fraction (4%) of V1a-expressing GABAergic neurons in the CeM projecting to the dorsolateral striatum determines female-specific anxiety state after social isolation (François et al., 2025).

**Figure 2:**
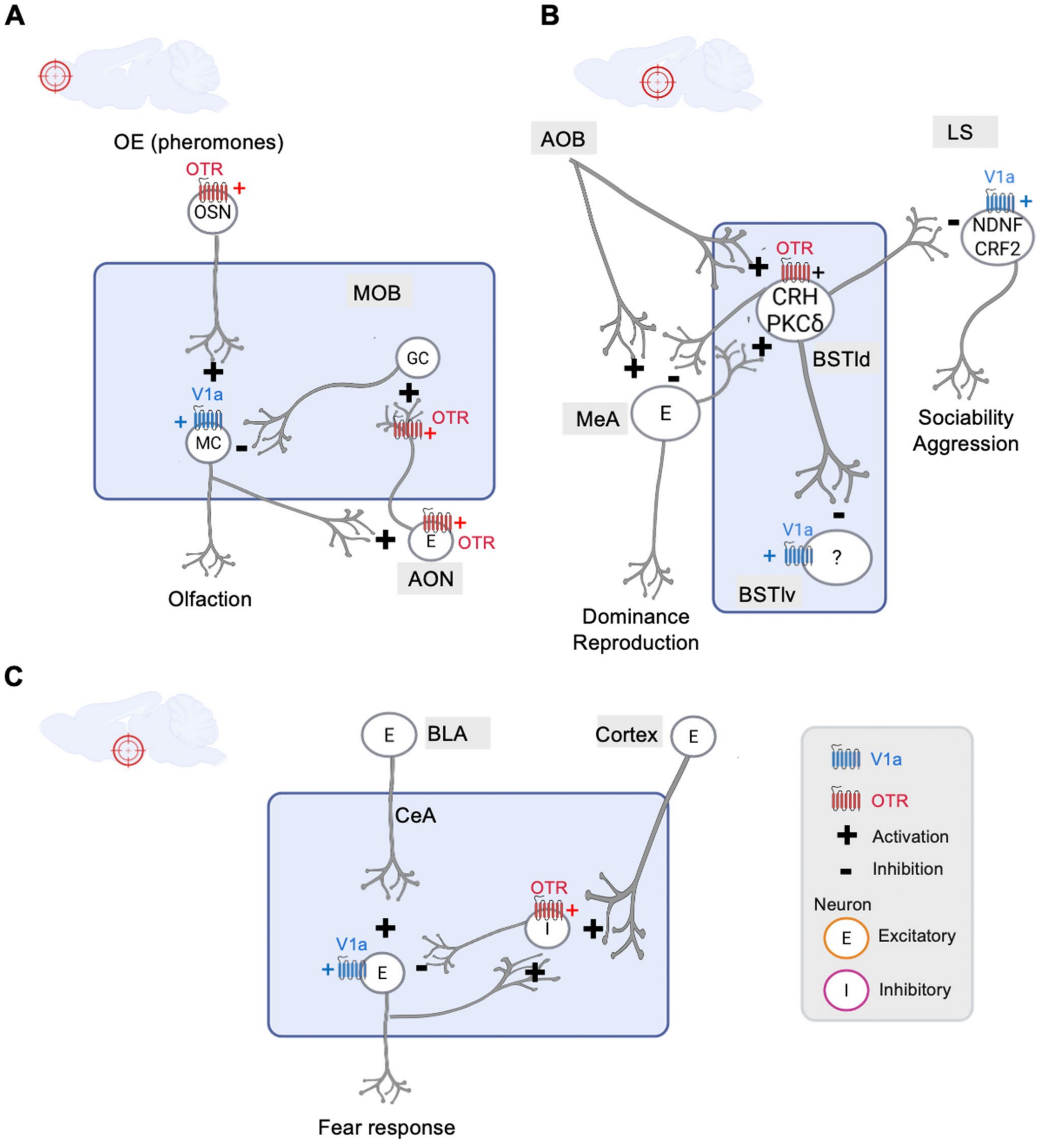
Circuit motifs modulated by OT/VP in the olfactory emotional systems. **(A)** Oxytocin (OT), via oxytocin receptors (OTR) facilitates neurotransmission from olfactory sensory neurons (OSN) to mitral cells (MC) where vasopressin (VP) via V1a signaling further facilitates this response. Consequently, further transmission to the anterior olfactory nuclei (AON) is juxtaposed with OTR-mediated AON feedback onto granule cells (GC)—suppressing MC activity in main olfactory bulb (MOB). This circuit motif is accelerated by OTR/V1a and at times, filtered by OTR. The source of OT and VP is diverse—volatile like in urine in the olfactory epithelium (OE), but from hypothalamic projections in the olfactory bulb (OB). **(B)** OTR facilitates inhibitory (I) transmission of CHR/PKd-positive inhibitory neurons in the bed nucleus of stria terminalis (BNST) laterodorsal onto medial amygdala (MeA) excitatory (E) neurons relaying olfactory signals, and lateral septum (LS) NDNF/CRF2-expressing inhibitory neurons, and uncharacterized V1a-expressing neurons in the lateroventral BNST. V1a facilitates excitatory inputs onto these neurons whereas OTR facilitates inhibitory inputs balancing excitation vs. inhibition with OT/VP. The sources of OT/VP in MeA, BNST, PVN and SON determine the flow of information through the circuit motif for modulating aspects of sociability, aggressivity, dominance and reproduction. **(C)** V1a facilitates excitatory transmission from basolateral amygdala (BLA) into central amygdala (CeA) that drives fear response. Collateral axons stimulate the inhibitory (I) brake activated by cortical afferences. OTR amplifies this inhibitory tone, forming a circuit motif with separate nodes actionable by OT/VP coming from PVN. Created with Biorender.com.

The juxtaposition of two opposing modes of action, indirect inhibition by OT and direct excitation by VP, creates a *push-pull dynamic,* which could enact rapid and flexible modulation of threat responses (Figure 2C). Crucially, the interaction between OT and VP within the CeA would not be simply additive but structured by the connectivity of the underlying circuit. The inhibitory projections from CeL to CeM neurons mean that OT activation can suppress the activity of neurons that VP seeks to excite. This establishes a competitive dynamic in which the relative levels of OT and VP signaling would determine the net output of the circuit. Such a mechanism allows for context-dependent regulation, where internal states or external cues bias the system toward either defensive or affiliative responses (Stoop, 2012).

This microcircuit logic may extend beyond the CeA to the BNST, which mediates sustained anxiety states. While the CeA is primarily involved in phasic, immediate responses to acute threats, the BNST is implicated in prolonged states of apprehension and uncertainty (Flanigan and Kash, 2022). Notably, the distribution of OTR and V1a within the BNST mirrors the complementary pattern observed in the CeA, suggesting that similar push-pull dynamics may operate across these regions (Rigney et al., 2024; Francesconi et al., 2025). Indeed, OT has been shown to exert excitatory effects in specific BNST subregions increasing arousal and anxiety (Duque-Wilckens et al., 2018), whereas VP and OT signaling at OTR synapses reduces anxiety (Francesconi et al., 2025), supporting the notion of a conserved antagonistic architecture across the extended amygdala (Figure 2B).

The functional implications of this organization are profound. By embedding opposing neuromodulatory influences within a single interconnected circuit, the brain could achieve a high degree of flexibility in behavioral output. Rather than relying on separate pathways for distinct behavioral states, the same circuit can be tuned toward different outcomes through modulation of its internal balance at multiple anatomical sites like the BNST and CeA—determined by the balance of afferent and efferent projections. This arrangement is particularly advantageous in environments where rapid switching between states of vigilance and social engagement is required.

Importantly, afferences to this extended amygdala network from the hippocampus, lateral septum, prefrontal cortex, and hypothalamus shape the output of OT/VP modulation. Inputs from the ventral hippocampus, for example, convey contextual information about the environment and can modulate CeA activity during fear learning and extinction. OT-mediated enhancement of inhibitory tone within the CeA may facilitate the suppression of fear responses in safe contexts, whereas VP signaling may reinforce threat associations during learning (Stoop et al., 2015). This interplay suggests that OT and VP not only regulate immediate behavioral responses but also influence the encoding and retrieval of emotional memories (further discussed in section 5).

### 3.3 Factors affecting OT/VP action

The expression motifs of the receptors are influenced by developmental and experiential factors (Arakawa et al., 2010; Caughey et al., 2011). Early-life stress, for instance, has been shown to alter the expression of OTR and V1a, as well as the functional connectivity of amygdala circuits (Lesse et al., 2017; Schmidt et al., 2018; Oliveira et al., 2019). Such changes could disrupt the push-pull dynamics between OT and VP, thereby increasing vulnerability to anxiety disorders and maladaptive stress responses later in life. Epigenetic mechanisms, including DNA methylation of receptor gene promoters, are thought to play a key role in mediating these long-lasting effects (King et al., 2017).

Moreover, sex differences in OT/VP signaling further shape the function of these circuits (Lukas and Neumann, 2014; Dumais and Veenema, 2016; Rigney et al., 2019). Females generally exhibit higher baseline OT levels and greater OTR expression in limbic regions, which may confer enhanced resilience to stress and promote social bonding. In contrast, males often show stronger VP signaling in regions associated with aggression and territorial behavior. These differences are influenced by gonadal hormones, particularly estrogen, which upregulates OTR expression and modulates receptor sensitivity. As a result, the balance between OT/VP signaling, and thus the functional output of the extended amygdala, can vary significantly across sexes and hormonal states.

The integration of OT/VP signaling within the extended amygdala thus represents a paradigmatic example of how neuromodulators can shape circuit function through both *cooperative* and *competitive* interactions. By acting on distinct but interconnected astro-neuronal populations, these peptides implement a dynamic regulatory system capable of fine-tuning behavioral responses to complex and changing environments.

While the above body of evidence is generated mainly from classical auditory/associative fear conditioning and anxiety tasks, exceptions do exist. OT could differently modulate aversive and non-aversive output via the same brain region. In the anterior cingulate cortex, for example, action of OT enhances freezing response in observational fear conditioning (Pisansky et al., 2017), while OT action in the same region reduces pain in a sciatic nerve injury model (Esmaeilou et al., 2022). A more critical experimental investigation of local OT/VP microcircuits in cortical circuits could provide further clarity on the opposing behavioral responses posited by the same neuropeptide.

## 4 Processing attachment via discrimination: modulation in olfactory salience extended systems

### 4.1 OT/VP receptor expression

In contrast to the antagonistic dynamics observed in emotion-processing circuits, OT and VP often act in a *coordinated* and *synergistic* manner within neural systems dedicated to social signal detection and recognition. The olfactory system, particularly in species that rely heavily on chemosensory communication, provides a clear example of how these neuropeptides converge to enhance the salience and persistence of socially relevant stimuli (Wacker and Ludwig, 2012). The main and accessory olfactory bulbs constitute the first central relay for olfactory information, receiving input from the olfactory epithelium and vomeronasal organ. Within these structures, mitral and tufted cells serve as the principal output neurons, transmitting processed sensory information to higher-order regions such as the piriform cortex and amygdala. The activity of these output neurons is tightly regulated by local interneurons, particularly granule cells, which provide inhibitory feedback through dendrodendritic synapses. This reciprocal circuitry enables the refinement of sensory representations and the modulation of signal-to-noise ratio.

VP enhances the excitability of mitral cells in the olfactory bulb, thereby increasing their responsiveness to incoming sensory input (Tobin et al., 2010). One correlate is that VP effectively amplifies the representation of olfactory stimuli, particularly those associated with social cues by increasing the gain of output neurons through V1a receptor activation and modulation of intrinsic membrane properties as well as synaptic transmission (Tobin et al., 2010).

OT, on the other hand, acts primarily to reduce inhibitory input onto mitral cells. This disinhibitory effect is achieved through presynaptic modulation of granule cell activity, leading to a decrease in GABA release onto mitral cells (Osako et al., 2001; Fang et al., 2008; Oettl et al., 2016). The result is a further enhancement of mitral cell output, not by increasing excitation directly, but by relieving inhibitory constraints. This complementary mechanism allows OT to selectively boost the transmission of salient signals without broadly increasing network excitability.

### 4.2 Circuit function

The combined action of VP and OT within this circuit results in a powerful *excitatory amplification* of social olfactory signals (Figure 2A). Consequently through downstream circuits, both peptides enhance social memory and odor recognition, with their combined action extending the temporal window of recognition (Dluzen et al., 1998; Ferguson et al., 2000). This synergistic interaction reflects a broader principle in neuromodulation that multiple modulators can converge on the same circuit to achieve a common function through distinct mechanisms. In this case, enhancing excitation and suppressing inhibition allows for a two-factor enhancement of signal processing, enabling animals to rapidly detect and respond to socially relevant cues.

**Figure 3:**
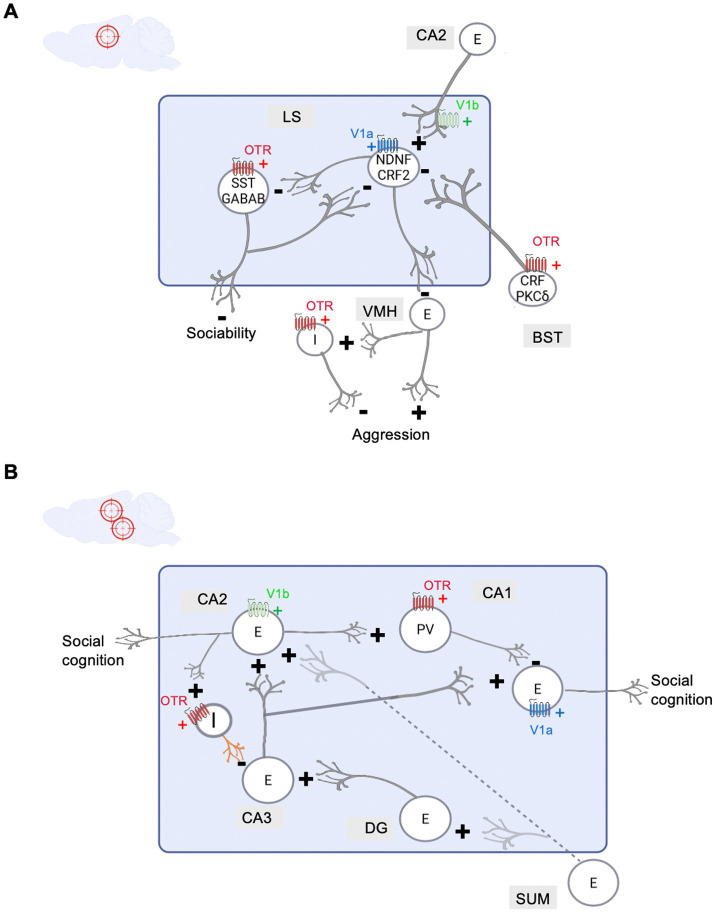
Circuit motifs modulated by OT/VP in the hippocamposeptal axis. **(A)** V1a and V1b facilitate excitatory transmission from hippocampal CA2 subfield onto Neuron-derived neurotrophic-factor/Corticotropin releasing factor-2 (NDNF/CRF2)-expressing neurons in LS by distinct mechanisms (postsynaptic vs. presynaptic) depending on the dose of local VP. OTR amplifies inhibition at monosynaptic projection somatostatin (SST)-expressing neurons in LS disinhibiting sociability, and at secondary projection inhibitory neurons in ventromedian hypothalamus (VMH) excited by aggression-promoting cells. Projections coming from the BNST neurons expressing Corticotropin releasing factor (CRF/OTR) suppress CRF2/V1a neurons that prevents disinhibition of sociability and promotes aggression. Cognitive function may override BNST inhibitory brake by actioning V1b excitatory signaling at presynaptic terminals stimulating the CRF2 neurons. Here, OT/VP from the SON, PVN and BNST balance disinhibition along circuit motifs to prioritize defensive vs. offensive motivated behaviors. **(B)** Social recognition and discrimination relies on hippocampal circuit motifs between subfields and projecting outside to numerous limbic structures notably via excitatory (E) CA2 neurons expressing V1b and CA1 neurons expressing V1a. OTR facilitates inhibitory (I) transmission in CA3 and CA1 via parvalbumin (PV)-expressing neurons balancing the excitatory drive amplified by VP signaling. Extrahippocampal excitatory inputs from supramammillary (SUM) nucleus exploits these circuit motifs to regulates social cognition. The sources of OT/VP are PVN, SON, and accessory hypothalamic nuclei. Created with Biorender.com.

The influence of OT and VP extends beyond the olfactory bulb to downstream regions involved in social behavior, including the medial amygdala and lateral septum (LS). These regions integrate olfactory information with hormonal and contextual signals to guide appropriate behavioral responses (Tendler and Wagner, 2015; Zylbertal et al., 2017; Mohapatra et al., 2024). Within these circuits, the balance between OT and VP signaling can shape social preferences, aggression, mating behaviors, and parental care (Smith et al., 2019). Electrophysiological studies show that VP excites a subset of GABAergic neurons synapsing onto somatostatin (SST) interneurons via postsynaptic V1a and presynaptic V1b in glutamatergic inputs from CA2 (Borie et al., 2021a). OT directly inhibits SST neurons via OTR signaling through GABA-B receptors, further enhancing the inhibitory tone or reshaping network synchrony via axon collaterals synapsing back onto V1a-expressing neurons (Borie et al., 2024). Such circuit motif allows the expression of opposing behaviors through disinhibition of downstream parallel projection pathways for controlling aggression (facilitated by VP), sociability (facilitated by OT) or anxiety (facilitated by OTR chemogenetic activation; Wong et al., 2016; Huang et al., 2021; Dromard et al., 2024). Remarkably, a cellular population in the LS expresses both OTR and V1a, suggesting that other mechanisms exist to integrate OT/VP functions (Hartswick et al., 2025). A similar population co-expressing OTR and V1b exists in CA2 neurons projecting to LS for *excitatory amplification* (Leroy et al., 2018), and CA3 targets for *feedforward inhibition* (Cymerblit-Sabba et al., 2023). Similar circuit motifs with V1a/OTR nodes of regulation were identified in the BNST cells projecting to LS (Rigney et al., 2024; Francesconi et al., 2025; Hartswick et al., 2025): VP disinhibits, OT excites CRF/OTR/PKC∆ inhibitory cells projecting in the LS to presumably V1a/CRF2-positive inhibitory cells. The latter receives feedback inhibition by OTR/SST-expressing inhibitory LS cells and predicted excitatory CA2 projections from principal V1b cells (Figure 3A).

### 4.3 Factors affecting OT/VP action

Timing is a key component of OT/VP regulation in circuit motifs. Recording of theta-frequency oscillations in the LS further indicated that OT effects lasted 10 min whereas VP effect extended beyond 60 min (Borie et al., 2021a), a differential timing attributed to the *excitatory amplificatory* gain by VP that contrasts with the *inhibitory brake* of OT in the LS. This could have implications for the modification of oscillatory pattern generation throughout the hippocamposeptal axis (discussed in section 6). Interestingly, the timing of peptide releases is critical to operate the directionality of OT/VP circuit motifs in the septum. VP is released in the LS during social novelty whereas OT is secreted during social familiarization, thereby providing sequence order to presentation of both peptides (Borie et al., 2024). VP alone or timely released with OT inhibit SST/OTR neuron outputs while preserving V1a/CRF2 neuron outputs (Figure 3A). However, OT alone inhibits both SST neurons outputs and V1a-expressing input cells via collateral inhibition. Remarkably, optogenetic control that generated a reverse sequence order (OT first, VP second) caused abnormal social familiarization and discrimination (Borie et al., 2024). Both peptides converge as inhibitory onto SST neurons: V1a signaling via GABA-A synaptic transmission from predicted CRF2-positive cells and OTR via GABA-B, confirming the existence of *feedforward inhibition*.

The combination of ligands and OT/VP microcircuit organizes a powerful computational motif that can run *synergistic amplification* or *antagonism*. It is believed that such functional units and their modulators allow circuits to encode opposing valence states (safety vs. threat) within the same anatomical structure (Besnard and Leroy, 2022). Such engines are thus fed by the source of OT and VP mapped in the SON when the animal transitioned between social fear-to-safety or in the PVN when transitioning between social safety-to-fear (Chakraborty et al., 2024). Additionally, OT/VP source differs on the valence of the experience—from the paraventricular nucleus PVN (when transitioning from safety-to-threat), the BNST (safety-to-anticipatory threat), and the supraoptic nucleus SON (threat-to-safety; Borie et al., 2021a; Oliveira et al., 2021; Dromard et al., 2024; Hartswick et al., 2025). Both peptides converge as inhibitory inputs onto SST neurons: V1a signaling via GABA-A synaptic transmission from CRF2 cells and OTR via GABA-B, confirming the existence of *feedforward inhibition*. Although VP neurons reside in the BNST, functional sources of OT and VP were mapped in the suprachiasmatic nucleus, PVN and SON (anticipatory anxiety-threat avoidance; Francesconi et al., 2025). However, alternate pathways exist as another circuit motif that bypasses the LS directly controls OTR-expressing neurons in the ventromedial hypothalamus (VMH) from the OT neurons in the retrochiasmatic supraoptic region (threat-to-avoidance; Osakada et al., 2024). Moreover, VP anti-serum injected in the LS or in hippocampus blocked social recognition in rats similar to OT anti-serum injected in hippocampus (but not in LS), suggesting possible species differences or protocol difference (2 repeat interactions to discriminate between unknown rat juveniles; Van Wimersma Greidanus and Maigret, 1996) contrary to 5 repeat interactions to discriminate between novel and familiar mouse juveniles (Borie et al., 2024). Despite the inconsistency, methodological differences confirm that OT signals in dorsal LS during familiar encounters but not novel encounters contrary to VP signaling.

Sex, age and experiential factors play a critical role in shaping these systems (Dumais and Veenema, 2016). Additive effects of OT and VP are not uniform and can be tuned to specific stimuli, such as those associated with conspecifics or reproductive cues. Lactation in females is accompanied by higher OTR levels in the LS that bias the septal microcircuit with V1a-expressing neurons, resulting in disinhibited aggressiveness toward peers (Menon et al., 2018). Consistently, injection of OT or VP reduces aggressivity of females expressing lower OTR and V1a in the LS while antagonism of septal V1a and OTR induces aggression, which supports the existence of a parallel-projection pathways as a functional unit balancing the expression of opposing behaviors (Oliveira et al., 2021). Early-life exposure to social stimuli can influence the expression of OTR and V1a in olfactory and limbic regions, thereby modulating the sensitivity of these circuits to social cues (Lukas et al., 2010). Disruptions in this process, such as those caused by early neglect or stress, can lead to long-lasting deficits in social recognition and affiliative behavior (discussed in section 9).

Taken together, the olfactory discrimination system exemplifies how OT and VP can act in *concert* to enhance the processing of socially relevant information, in contrast to their *opposing* roles in threat-related circuits. This duality underscores the versatility of these neuropeptides and highlights the importance of circuit context in determining their functional effects.

## 5 Processing cognition: modulation in memory systems

OT and VP also exert profound influences on learning and memory systems. These effects are particularly evident within the hippocampus and its associated limbic circuitry, where both peptides modulate synaptic transmission, network oscillations, and long-term plasticity (Cilz et al., 2019). Notably, in contrast to the clear *push-pull antagonism* observed in threat-processing circuits, the interaction between OT and VP in memory systems is more nuanced, involving both complementary and temporally dissociable mechanisms.

### 5.1 OT/VP receptor expression

The hippocampus exhibits a striking segregation of receptor expression, with OTR localized in inhibitory SST and parvalbumin (PV) neurons of CA1 and subiculum, in CAMKII-positive excitatory cells of CA2/CA3, V1a enriched in inhibitory neurons of CA1, while V1b is exclusive in excitatory neurons of CA2 (Owen et al., 2013; Leroy et al., 2018; Tirko et al., 2018). Although these patterns vary between species and strains, segregation between receptor subtypes is maintained, confirming that the OT/VP systems are balanced. This anatomical organization suggests that OT and VP may differentially influence distinct stages of information processing within hippocampal circuits and its inputs from the entorhinal cortex (Leung et al., 2018). In particular, CA3 is widely regarded as a key locus for pattern completion and associative memory retrieval, whereas CA1 integrates processed information and relays it to cortical and subcortical targets (Lin et al., 2018).

### 5.2 Circuit function

At the cellular level, OT exerts a rapid, transient (*about ten minutes*; Bohus et al., 1978; Galbusera et al., 2017) and reversible modulatory effect primarily through excitation of GABAergic interneurons. In CA1, activation of OTR depolarizes interneurons located within the *stratum pyramidale* and *stratum oriens*, leading to increased inhibitory tone onto pyramidal neurons. This mechanism results in a net suppression of pyramidal cell firing, effectively sharpening signal transmission by reducing background noise and enhancing the signal-to-noise ratio of synaptic inputs (Owen et al., 2013). In CA2, OT transforms the firing mode of principal cells (Tirko et al., 2018). Such modulation is thought to stabilize network activity and may contribute to the gating of information flow during memory encoding (Tirko et al., 2018; Cilz et al., 2019; Zhang et al., 2024).

In contrast, VP has been associated with rapid longer-lasting effects on synaptic plasticity via V1a and V1b in CA1 and CA3, respectively, including facilitation of long-term potentiation (LTP) and memory consolidation (Chepkova et al., 1995; Galbusera et al., 2017). These effects are often observed at low, physiologically relevant concentrations and can persist well beyond the period of peptide application (one hour; Bohus et al., 1978). Unlike the acute modulation mediated by OT, VP-induced plasticity does not necessarily involve direct changes in neuronal excitability but rather enhances synaptic efficacy and strength through intracellular ERK-CREB signaling (Pagani et al., 2015). Enhanced CA2 transmission by V1b (increased EPSPs), or activity by OTR (burst firing in CA2), is predicted to suppress CA3 via backpropagating feed-forward inhibition that shifts subfield activity pattern. Both work in concert to promote this burst firing of CA2 principal cells that project to CA1 for supporting social memory (Leroy et al., 2018; Cilz et al., 2019).

*In vivo,* CA2 synapses regulate aggression and social memory notably via V1b that promotes excitation of projections to PV cells in CA1 and also into the septum (Pagani et al., 2015; Leroy et al., 2018; Meira et al., 2018). CA2 neurons receiving inputs from the supramammillary nucleus process social novelty, in contrast to collaterals going to the dentate gyrus that process contextual non-social novelty (Chen et al., 2020). Thus, regulation of social vs. non-social recognition depends on circuit distribution along the dorso-ventral and antero-posterior axes (Raam et al., 2017) and subfield specific input projections from the hypothalamus (Chen et al., 2020). The preferential targeting by VP and OT of discrete neuronal subsets within these subregions provides a structural basis for their complementary roles in social memory (Chevaleyre and Siegelbaum, 2010; Grinevich and Stoop, 2018). The effects of OT/VP are expected to be *concerted* in CA3 → CA2 projections, both enhancing excitation but *opposite* in CA2 → CA1 projections due to V1b/CAMKII cells stimulatory relay by OTR/PV cells or direct projections to modulate V1a/CAMKII neurons in CA1 (Figure 3B).

Furthermore, the temporal divergence in the dynamics of OT/VP action suggests a division of labor between these neuropeptides in memory processes. OT may primarily regulate the immediate encoding and filtering of information, ensuring that salient inputs are selectively processed, whereas VP may support the stabilization and consolidation of these representations over longer timescales (Urban and De Wied, 1986; Chepkova et al., 1995). Importantly, these processes are not independent but interact within the same network for modulating timing-dependent plasticity that coordinates memory formation and retrieval (Leroy et al., 2019).

The influence of OT and VP on hippocampal function extends beyond synaptic plasticity to the regulation of network oscillations (Bohus et al., 1978; Urban, 1981, 1998; Chepkova et al., 1995; Borie et al., 2021a; Zhang et al., 2024). Oscillatory activity, particularly in the theta and gamma frequency ranges, is thought to be critical for coordinating neuronal ensembles during memory encoding and retrieval (Mohapatra et al., 2024). By modulating inhibitory interneurons, OT influences the timing and synchronization of these oscillations in *competition*, and at times in *cooperation* with acetylcholine, to shift the temporal precision of neuronal firing that brings about network state transitions (Zhang et al., 2024). VP, through its effects on synaptic plasticity and excitatory transmission, may further shape the stability and persistence of oscillatory patterns (Bohus et al., 1978).

### 5.3 Factors affecting OT/VP action

The hippocampus does not operate in isolation but is part of a broader network. OTR, V1a and V1b are expressed along large-scale neuron networks that include the hippocampo-septal axis and its hypothalamic and brainstem projections (Rizzi-Wise and Wang, 2021; Besnard and Leroy, 2022), where they have shown *concerted* and *opposite* effects on social memory and affiliative behaviors depending on local and distributed networks (Leroy et al., 2018; Cymerblit-Sabba et al., 2023; Borie et al., 2024; Dromard et al., 2024). At the microcircuit level, VP excites a subset of V1a GABAergic cells (and indirectly via V1b CA2 inputs; Joëls and Urban, 1984; Leroy et al., 2018) expressing the markers Corticotropin releasing factor receptor-2 (CRF2) and Neuron-derived neurotrophic factor (NDNF) to consequently suppress OTR neurons-expressing somatostatin (SST). This is predicted to remove the disinhibition of V1a/CRF2 via backpropagating fibers. Such an example of *concerted disinhibition* would require timed release of OT/VP to transform circuit output through both V1a/CRF2 and OTR/SST parallel outputs.

Given that V1b expressing CA2 pyramidal neurons project to the LS, we predict modulatory Influences of such hippocampal projections on *push-pull interaction* of OT/VP in the septum. As explained above, VP agonism followed by OTR activation in the LS supports social discrimination that a reverse sequence impedes (Lukas et al., 2011, 2013; Borie et al., 2024)—possibly due to temporal dynamics of OT/VP releases by distinct contexts and duration of its electrophysiological effects (shorter for OT, longer for VP; Van den Hooff and Urban, 1990; Urban, 1998). Physiologically, this aligns with LS OT/VP secretions cued to the degree of social-cue familiarity (VP by strangers vs. OT by mates) and its sources (PVN if negative vs. SON if positive vs. BNST if anticipatory; Borie et al., 2021a, 2024; Dromard et al., 2024). The interplay between OT and VP in such inhibitory microcircuits reflects a complex integration of acute neuromodulation and long-term plasticity (Bohus et al., 1978; Urban, 1981, 1998; Urban and De Wied, 1986; Chepkova et al., 1995; Grinevich and Stoop, 2018; Besnard and Leroy, 2022; Mohapatra et al., 2024). Feedback loops may also extend beyond the hippocamposeptal axis to hypothalamus and its projections back in the hippocampus to regulate OT release and inhibitory patterning (Zhang et al., 2024). By acting on distinct components of the network, these peptides can coordinate the encoding, consolidation, and retrieval of social memories. Again, sex, age and species variations in regional expression should be taken into consideration when designing experiments, given their roles in influencing and dictating OT/VP function (Cilz et al., 2019).

## 6 Processing internal states: modulation in autonomic systems

### 6.1 OT/VP receptor expression

OT/VP signaling also regulates internal physiological states that support adaptive responses (alert vs. homeostasis). Within brainstem circuits, OT/VP from the PVN and SON exert *opposing* and *concerted* effects that balance (para)sympathetic activities in response to environmental and internal demands. The nucleus of the solitary tract (NTS), dorsal motor nucleus of the vagus nerve (DMN), the parabrachial nucleus (PBN), hypoglossal nucleus (HPN), the pre-Botzinger complex (PrBo) and rostral ventrolateral medulla (RVLM) form the core of a central autonomic network that expresses OTR, V1a and V1b (Zhao and Ai, 2011). These structures integrate visceral sensory information, fluid balance, satiety and generate appropriate motor outputs to maintain homeostasis. They are also tightly interconnected with limbic regions such as the amygdala and hypothalamus, enabling emotional states to influence motivated behaviors intended to adapt physiological responses (drinking, feeding, breathing, heart rate).

### 6.2 Circuit function

**Figure 4:**
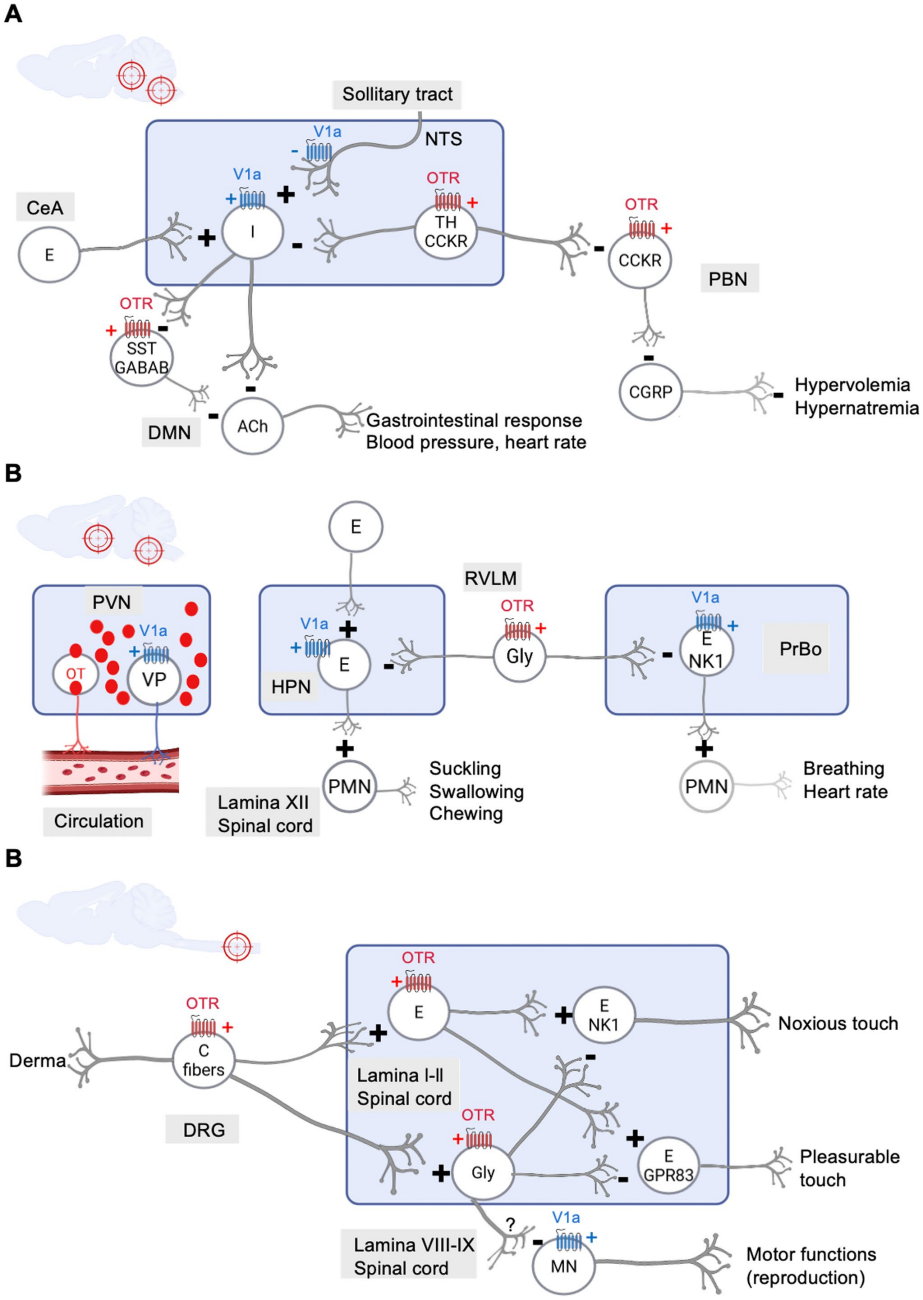
Circuit motifs modulated by OT/VP in brainstem and spinal cord. **(A)** Sensory ascending fibers from peripheral organs bundled in the solitary tract stimulate inhibitory (I) neurons in the nucleus of the solitary tract (NTS) that V1a suppress via presynaptic mechanism, and at times, facilitate excitatory projections from amygdala. These neurons use direct projections to cholinergic (ACh) neurons in the dorsal motor nucleus (DMN) and indirect projections via neurons expressing somatostatin (SST) and GABA_B_, forming a feedforward disinhibitory relay actionable by OTR signaling to regulate heart rate, blood pressure, gastrointestinal movement. OTR exert inhibitory actions on V1a cell afferences via NTS neurons expressing tyrosine hydroxylase/cholecystokinin receptor (TH/CCKR). These neurons also project to the parabrachial nucleus (PBN) to disinhibit Calcitonin gene-related peptide (CGRP)-expressing neurons that prevent hypovolemia and hypernatremia. OTR signaling amplifies this disinhibitory switch together with cholecystokinin (CCK) co-released with OT from PVN. **(B)** V1a facilitates excitation in hypoglossal nucleus (HPN) as in the pre-Bötzinger complex (PrBo) that both stimulate preganglionic premotor neurons (PMN). OTR facilitates inhibition of glycinergic (Gly) neurons in the rostral ventrolateral medulla (RVLM) and that project onto V1a-expressing excitatory (E) neurons of HPN and PrBo. Therefore, OT and VP from the paraventricular nucleus (PVN) and supraoptic nucleus (SON) counterbalance the HPN and PrBo outputs excitation onto premotor neurons functions. At times, somatodendritic release of OT at high concentration cross-reacts with V1a expressed in VP neurons to shape peptides secretion into the circulation. OT/VP from both blood and synaptic release coordinate sensorymotor functions by targeting both peripheral organs and its innervation modulate sucking, feeding, chewing, breathing and heart rate variability. **(C)** V1a and OTR segregated along a dorsoventral axis in the spinal cord amplify motor and sensory information, respectively. Both are interrelated by hypothetical inhibitory relays amplified by OT coming from the PVN and circulation. OTR facilitates excitation in sensory fibers and ascending pathways converging on excitatory neurons expressing Neurokinin-1 (NK1) or G-protein-coupled-receptor-83 (GPR83). Created with Biorender.com.

Within the autonomic nervous system, OT acts predominantly to enhance parasympathetic activity. In the DMN, OT inhibits OTR/SST neurons possibly via GABA-B signaling (Cruz et al., 2019) that sustains depolarization of preganglionic cholinergic neurons, increasing vagal output to the heart and gastrointestinal system (Figure 4A). In the NTS, OT and cholecystokinin (CCK) stimulate projection OTR neurons in PBN relaying on Calcitonin gene-related peptide (CGRP) neurons to suppress hypervolemia and hypernatriemia (Ryan et al., 2017). Remarkably, a subset of PVN OT neurons co-expressing CCK projects to the NTS, and chemogenetic activation of these cells activated NTS OTR cells-expressing tyrosine hydroxylase (TH), suggesting the possible existence of a CCK-inhibitory brake on OTR/TH cells. Remarkably, a loss of CCK sensitivity impairs satiation on a high fat diet (Gruber et al., 2023).

V1a directly excites half of NTS neurons, but at the same time, depresses the glutamatergic visceral afferents to these neurons by acting presynaptically, biasing the system toward sympathetic dominance (Raggenbass, 2008). A similar effect was detected in PBN, suggesting that the combination of CCK, OT, and VP likely contributes to a common mechanism. Briefly, shifting the inhibitory-excitatory balance to modulate neurotransmission along predisposed neuronal networks regulate functional output. These combined effects would prepare the organism for action in the face of potential threats by decreasing heart rate, enhancing digestive activity, and a physiological state conducive to rest and social engagement. The *opposing* effects of V1a and OTR in DMN and PBN are consistent with the filtering by dissociation of the signals transiting from the solitary tract and those from the amygdala.

In the HPN, OTR has been shown to use KCC2 signaling to switch GABA to excitatory on principal V1a neurons that project to the premotor lamina XII for controlling tongue muscles (Wrobel et al., 2010). V1a also stimulates HPN principal cells that project on premotor neurons, suggesting local *amplificatory gains* mediated by OT/VP (Figure 4B). This response is amplified in the first days after birth to command swallowing, sucking, chewing, vocalization and breathing (Bolte et al., 2023).

In the RVLM, VP increases the excitability of neurons that project to spinal preganglionic neurons, thereby elevating blood pressure, heart rate and inspiratory bursting (Kc and Dick, 2010; Bolte et al., 2023).

In PrBo, OTR enhances the glycinergic (Gly) inputs of cardiac-innervating preganglionic neurons in the *nucleus ambiguus* for increasing heart rate variability and decreasing inspiratory rhythm during calming behaviors (Buron et al., 2025). It is possible that V1a neurons-expressing Neurokinin receptor-1 (NK1) receive direct inhibitory projections from OTR/Gly neurons thereby forming another example of circuit motifs balancing OT/VP actions (Kc et al., 2002).

What sources release OT/VP in these circuits? A major input is from the hypothalamic PVN (Kc et al., 2002; Buron et al., 2025), although coincident release into the circulation has been shown to contribute (Elsaafien et al., 2026). Remarkably, systemic or central V1a antagonism abolishes the increased blood pressure and heart rate after optogenetic excitation of OT neurons in the PVN. Therefore, multiple scales of regulation by OT and VP from receptor crosstalk to circuit motifs organize physiological responses.

The *opposing* and *concerted* actions of OT and VP within the autonomic network mirror their roles in emotional regulation. OT could promote a state of safety and social engagement, characterized by reduced arousal and increased affiliative behavior. VP, on the other hand, could enhance vigilance and readiness for defensive action. The coordination of these physiological and behavioral states is thought to be essential for adaptive functioning.

### 6.3 Factors affecting OT/VP action

Importantly, the balance between OT and VP signaling in autonomic circuits is not fixed but can be modulated by context, experience, and internal state. This is where other neuropeptides like CCK, CRF, NK1, glucagon (GCG) fit in this model. Stress, for example, can shift this balance toward increased VP activity, biphasic with OT activity, by gaining CRF sensitivity, leading to heightened sympathetic tone and reduced parasympathetic function (Nazarloo et al., 2025). Chronic stress may result in a persistent imbalance, contributing to the development of anxiety disorders, cardiovascular diseases, and other stress-related conditions. Impaired CCK sensitivity could alter the OT/VP balance in PVN projections to brainstem nuclei and lead to lack of satiation when desperately needed because the sole source of food is noxious (Gruber et al., 2023). This pathway might intersect with the previously identified nodose ganglia→NTS → LS pathway, in which OTR ganglia neurons excite NTS GCG neurons projecting on LS inhibitory cells expressing GCG-like peptide-1 receptor (GLP1R) to decrease motivation to feed (Terrill et al., 2019; Brierley et al., 2021). Several nodes of this network can interact with CRF and contributes to stress-induced hypophagia (Terrill et al., 2018).

The integration of OT/VP signaling across limbic and autonomic systems underscores the importance of these peptides in linking emotional and physiological responses. By modulating both neural circuits and peripheral organs, OT and VP enable coordinated adaptation of motivated behaviors to complex environmental challenges (food poisoning, thirst).

## 7 Processing sensorimotor functions: modulation in spinal cord

### 7.1 OT/VP receptor expression

The influence of OT and VP extends to spinal sensorimotor processing predict yet another dimension of their *coordinated* and *opposing* functions. Current evidence, however, does not support a strict anatomical segregation between OT and VP receptors along dorsoventral spinal domains. Descending projections from hypothalamic neurons, particularly those originating in PVN, predominantly target the superficial dorsal horn as well as autonomic regions of the spinal cord, and that both OT- and VP-containing neurons contribute to these projections (Sawchenko and Swanson, 1982; Cechetto and Saper, 1988; Caffé et al., 1989; Hallbeck et al., 2001; Eliava et al., 2016). Consistently, receptor mapping studies indicate that OTR binding sites are enriched in laminae I-II and V-VI of the dorsal horn (Eliava et al., 2016; Martínez-Lorenzana et al., 2021), whereas V1a display a broader distribution across spinal regions rather than a selective enrichment in ventral motor territories (Tribollet et al., 1989; Liu et al., 2003; Schorscher-Petcu et al., 2010).

### 7.2 Circuit function

The functional divergence between OT and VP in the spinal cord is better explained by state-dependent recruitment than by fixed anatomical segregation. Indeed, both peptides exert modulatory effects on nociceptive processing that vary according to physiological conditions such as stress, hormonal state, or injury. OT is consistently associated with activation of descending analgesic pathways and spinal inhibition of nociceptive transmission, notably through recruitment of inhibitory interneurons and modulation of glutamatergic inputs (Condés-Lara et al., 2006; Juif and Poisbeau, 2013; Eliava et al., 2016).

OT seems to exert a potent inhibitory effect on nociceptive transmission by at least three ways: at the periphery, acting on C-fibers, via OT release in the blood; centrally, and by direct axonal OT release at both the levels of the spinal cord and periaqueductal gray (PAG) by independent parvocellular populations (Eliava et al., 2016). In the spinal cord, by activating a subset of excitatory interneurons that, in turn, would recruit inhibitory GABAergic and glycinergic neurons, OT may enhance inhibitory control over incoming nociceptive signals (Figure 4C). *Inhibitory* and *excitatory* OTR cells reciprocally innervate NK1 cells and GPR83 cells that promote pain or reward, respectively. Excitation of GPR83 outputs by pleasurable social touch (massage) is amplified by excitatory OTR cells, while stimulation of NK1 cells by noxious touch is counteracted by inhibitory OTR cells (Bohic et al., 2026). This mechanism provides a first cellular basis for the well-documented analgesic effects of OT in the frame of social modulation of pain perception (Iwasaki et al., 2023). Complementarily, PVN → PAG OT projections are a second circuit by which OT exert its analgesic effect, in sex and modality independent manner (Iwasaki et al., 2023). Finally, the effects of OT seem to require GABA-B and TRPV1signaling (Gonzalez-Hernandez and Charlet, 2018; Fan et al., 2026) independently of V1a (García-Boll et al., 2018). Importantly, cross-reactivity between OT and VP systems further blurs this distinction, as OT-induced analgesia can be mediated, at least in part, through V1a signaling in primary sensory neurons (Schorscher-Petcu et al., 2010; Zheng et al., 2021).

In contrast, VP-mediated effects appear more context-dependent, displaying both anti- and pro-nociceptive actions depending on peptide concentration, receptor subtype engagement, and the organism’s physiological state (Koshimizu and Tsujimoto, 2009; Mogil et al., 2011; Juif and Poisbeau, 2013). VP directly excites motor neurons and premotor interneurons in the ventral horn. This excitation increases motor output and may facilitate behaviors requiring rapid or sustained muscle activity. VP can also modulate inhibitory interneurons by enhancing GABA-A function (Peng et al., 2015), adding a layer of complexity to its effects on motor circuits. This effect was reproduced by TGOT, a selective OTR analog indicating that an inhibitory interneurons sending projections in the ventral horn could be involved (Liu et al., 2003).

### 7.3 Factors affecting OT/VP action

Furthermore, it has been recently suggested that V1a expression in the spinal cord could be lateralized, mostly on the right side, with functional consequences on motor function (Bakalkin et al., 2026), a phenomenon that has previously been reported for OTR signaling in the left side of female auditory cortex (Song and Froemke, 2025). Lateralization adds to the other environmental and developmental factors that should be taken into consideration in study design.

The *coordinated* action of OT and VP in these systems rely on *parallel pathway segregation* (as discussed above) for the integration of sensory and motor functions in a context-dependent manner. For example, in a threatening situation, increased VP signaling may enhance motor readiness and suppress sensory distractions, whereas in a safe and social context, OT may reduce pain perception and promote relaxed motor activity—both these parallel outputs antagonistically regulating each other.

## 8 Astrocyte regulation by oxytocin and vasopressin

Rather than being a passive onlooker, astrocytes actively contribute to OT/VP signaling, adding complexity to the classical neurocentric view of their typical actions. Astrocytes in many brain regions express functionally active VP and OT receptors (Jurzak et al., 1995; Brinton et al., 1998; Tasker et al., 2012; Wahis et al., 2021; Baudon et al., 2025; Boi et al., 2026). OT/VP signaling in astrocytes triggers rapid activity-dependent structural plasticity which may allow a further dimension of modulation by both holding peptides distant from the time and site of release and lead to a local dynamic change in neuronal network excitability (Hatton et al., 1984; Miyata et al., 2001; Baudon et al., 2025). This is consistent with the often extrasynaptic or “volume-based” release of OT/VP that could shape the effective range, timing, and circuit specificity by controlling extracellular diffusion and neurotransmitter uptake. In PVN and SON, physiological shifts including lactation change structural plasticity of astrocytes, reducing coverage of magnocellular neurons, increasing extracellular diffusion and augmenting glutamate spillover that could engage crosstalk between glutamatergic and GABAergic inputs (Oliet et al., 2004; Piet et al., 2004). Astrocytic involvement in the long-term suppression of VP neuron firing by facilitation of its GABAergic inputs was demonstrated during hypoosmotic challenge. In conditions of high salt intake, VP signaling decreases astrocytic coverage of VP neurons by phagocytic microglia, again contributing on local osmotic regulation (Gu et al., 2025). In the cortex, V1a signaling increases astrocytic aquaporin-4 that boosts water flux in the parenchyma (Niermann et al., 2001). It should be noted that astrocytes directly respond to OT and VP, evidence for which came from hypothalamic cultures (Di Scala-Guenot et al., 1994; Zhao and Brinton, 2003). Astroglial OTRs produced a transient, dose-dependent rise in intracellular calcium due to release from intracellular stores (Di Scala-Guenot et al., 1994). Subsequent work showed that neurons can modulate astrocytic OT receptor expression in rat culture, with TGF-*β* and membrane components contributing to this regulation (Mittaud et al., 2002). In adult rat pituicytes, on the other hand, calcium is mobilized by VP via V1 receptors, while in isolated cortical/hippocampal astrocytes, VP increases both intracellular calcium and glutamate release (Hatton et al., 1992). VP therefore has the potential to recruit local astrocytes and thereby alter recurrent neuronal circuits.

Regulation of behavior by astrocytic activity has been demonstrated in the amygdala and hypothalamus and associated with structural remodeling of astrocytes as a function of social contexts such as stress, lactation or dehydration (Oliet et al., 2004; Baudon et al., 2025), perhaps to prolong cellular effects of those challenges on neuronal networks. In the CeA, morphologically distinct astrocyte subpopulation expresses OTRs, playing a role in OT-induced anxiolysis and positive reinforcement. In fact, activation of astrocytic OTR promoted excitation of local CeL neurons, altering amygdala output relevant to fear and comfort states (Wahis et al., 2021). In the PVN, on the other hand, OT regulates astrocytic gene expression, intracellular signaling, and proteins, both *in vitro* and *in vivo* (Meinung et al., 2025). In cultured rat astrocytes, OT causes rapid morphological plasticity dependent on ERK and OTR cascades, and eventual suppression of RhoA/ROCK signaling. *In vivo*, intracerebroventricular OT causes a rapid remodeling of GFAP-positive astrocytes, increasing astrocytic coverage. A specific knockdown of astrocytic OTR abolished the anxiolytic effects of local OT in the PVN without affecting locomotion, suggesting a causal role of OT-signaling in astrocytes in anxiety processes (Meinung et al., 2025). It has also been proposed that OT signaling coupling between neurons and astrocytes in the PVN is sexually dymorphic in the context of stress adaptation in mice (Sandoval et al., 2025). Gap junction via Connexin 30 seem to play an important role in coupling neuroglia OT signaling, notably in the PVN to sustain maternal behavior (Arnoux et al., 2026).

To date no studies investigated OT/VP complementary actions in astrocytes or astro-neuronal networks, warranting future interest in this area of research as it could uncover mechanisms underlying the diffusion and temporal dynamics of peptidergic signaling.

## 9 Dysfunction of oxytocin-vasopressin axis

Disruption of OT/VP signaling has been implicated in multiple neuropsychiatric disorders, including anxiety disorders, autism spectrum disorder (ASD), addiction, and depression (Neumann and Landgraf, 2012; Zhao et al., 2026). However, meta-analyses have not consistently identified global deficits in peripheral OT or VP levels, likely owing to methodological heterogeneity and the limited relationship between circulating peptide concentrations and central circuit function (Rutigliano et al., 2016; Reijnen et al., 2017; John and Jaeggi, 2021). Emerging evidence instead suggests that pathology may arise from dysregulation of OT/VP-dependent circuit motifs and their interactions with broader neural networks (Borie et al., 2021b).

### 9.1 Anxiety disorders and PTSD

*Preclinical evidence*. Hyperactivity of amygdala-centered threat circuits is coupled with reduced regulatory control from prefrontal and inhibitory systems. OT and VP exert largely opposing influences within amygdala-centered threat circuits. OT may recruit inhibitory microcircuits in the CeA and related structures, facilitating fear extinction and stress buffering, whereas VP signaling through V1a/V1b receptors generally enhances threat processing and stress responsivity. The evidence is consistent with abnormal complementary OT/VP actions within the *push–pull* organization of defensive circuits.

*Clinical evidence*. Post-traumatic stress disorder (PTSD) patients exhibit altered amygdala activity and elevated VP levels in some cohorts (Horn et al., 2024). Intranasal OT frequently reduces amygdala reactivity and enhances functional coupling with regulatory networks, although findings remain inconsistent across studies (Spengler et al., 2017; Kou et al., 2022). VP injection in healthy men does not reduce amygdala reactivity to fear *per se*, instead, it reduces connectivity between the amygdala and prefrontal cortex (Zink et al., 2010), hinting at stronger cortical inhibition. Together, available evidence supports a role for OT/VP imbalance in anxiety disorders (Feldman et al., 2014), but causal mechanisms remain unresolved.

*Future directions*. We predict that a combination of reduced OT-mediated inhibition and increased VP-mediated excitation, provides a mechanistic framework for explaining the amplification of fear responses and promotion of hypervigilance. Therapeutic strategies aimed at restoring the OT/VP balance may be more effective than simply enhancing one system alone. To this effect, combination therapy with OT analogs and VP antagonists (or other relevant peptides) is currently being assessed (Gupta and Prabhavalkar, 2021; Chaki, 2021).

### 9.2 Mood disorders

*Preclinical evidence*. VP signaling contributes to hypothalamic–pituitary–adrenal (HPA) activation and stress responsiveness specifically influences cognitive empathy, whereas OT is generally associated with social buffering and resilience primarily signaling emotional empathy in rodent models (Uzefovsky et al., 2015; Teixeira et al., 2025). Nevertheless, both excessive and deficient VP signaling can produce affective abnormalities, indicating that behavioral outcomes depend on circuit context and receptor balance rather than peptide abundance alone (Surget and Belzung, 2008; Csikota et al., 2016; Whylings et al., 2021).

*Clinical evidence*. Altered OT dynamics, OTR polymorphisms, and impaired social engagement have been associated with depressive disorders and postpartum depression (Apter-Levy et al., 2013; MacKinnon et al., 2014; Harrison et al., 2026). However, effect sizes are modest and findings vary across cohorts. Current evidence supports involvement of OT/VP systems in affective regulation but does not yet identify robust disease biomarkers.

*Future directions*. A recurrent theme is that pathology may arise from disrupted coordination between OT- and VP-sensitive networks rather than dysfunction of either system alone. This perspective shifts the focus from single-target interventions to strategies that consider network-level balance and context-dependent modulation.

### 9.3 Autism spectrum disorders

*Preclinical evidence*. ASD models reveal prominent OT/VP dysfunction within lateral septal and hypothalamic circuits. Impaired V1a signaling disrupts septal OTR/SST microcircuits, producing deficits in social discrimination and increased aggression. Restoration of OT and VP signaling together within these pathways rescues social and emotional phenotypes better than single peptide injection, highlighting the importance of coordinated peptidergic modulation rather than isolated receptor deficits (Borie et al., 2021a, 2024; Chakraborty et al., 2024; Dromard et al., 2024; Bortolozzo-Gleich et al., 2025). The evidence is consistent with abnormal complementary OT/VP actions within the synergistic amplification organization of social discrimination circuits.

*Clinical evidence*. Reduced cerebrospinal fluid VP levels correlate with social impairments in children with ASD (Oztan et al., 2020), and genetic variation in OTR and V1a has been associated with social phenotypes. However, clinical studies remain heterogeneous, suggesting that OT/VP dysfunction may characterize specific ASD subgroups rather than the spectrum as a whole, and causal mechanisms remain unresolved.

*Future directions*. Clinical heterogeneity between patients highlights the need for a more nuanced understanding of how OT/VP regulation interacts within specific circuits and how their balance is altered in different subtypes of the disorder (Clarke et al., 2024). Understanding how the OT/VP balance within specific circuit motifs contributes to the brain transitioning between different states could further explain clinical heterogeneity and contextual dependency. This hypothetical framework is supported by preclinical studies indicating that transitioning from social aversion-to-safety and vice-versa engages different source of OT/VP for filtering the flow of information across circuit motifs (Borie et al., 2024; Dromard et al., 2024). Anticipation by anxious arousal might also shift the source of peptides to redirect the flow of information through the broader social discrimination circuit motifs that integrate valence with salience (Borie et al., 2021a; Bortolozzo-Gleich et al., 2025).

### 9.4 Addiction

*Preclinical evidence*. OT and VP differentially regulate reward and stress circuits implicated in addiction (Zanos et al., 2014). OT reduces drug-seeking, withdrawal symptoms, and stress-induced reinstatement, whereas VP signaling, particularly through V1b receptors, facilitates relapse and excessive alcohol consumption (Skuse and Gallagher, 2009; Edwards et al., 2012; Song et al., 2016; Xiao et al., 2017). These effects likely emerge from interactions between peptidergic systems, mesolimbic dopamine pathways, and the extended amygdala.

*Clinical evidence*. Human evidence remains limited, results mixed (Rastogi et al., 2024). Neuroimaging studies suggest that OT modulates reward-related activity and social reinforcement, but clinical trials targeting addiction are still insufficient to establish efficacy. Translation of preclinical findings therefore remains an important challenge. OT associated changes in the epigenome are proposed as possible marker for relapse (Fan et al., 2021; Salles et al., 2024).

*Future directions*. We predict that the opposing of OT and VP on addiction-related behaviors reflect their broader roles in regulating stress and reward systems in which OTR, V1a and V1b are expressed, likely in distinct cell populations (Skuse and Gallagher, 2009). Importantly, the balance between OT/VP signaling may influence the neural substrate determining susceptibility to relapse, particularly under conditions of stress (Bowen and Neumann, 2017).

### 9.5 Emerging perspective

The hypothesis that context- and sex-specific effects depend on circuit motif organization shifts attention from OT/VP abundance toward receptor-defined microcircuits that integrate social, emotional, and stress-related information, and may provide a more useful framework for therapeutic development.

## 10 Therapeutics targeting the oxytocin-vasopressin axis

The therapeutic exploitation of OT and VP signaling remains challenging because both peptides exhibit short peripheral half-lives and limited brain penetration (Jurek and Neumann, 2018; Zhao et al., 2026). These limitations have stimulated the development of receptor-selective agonists, antagonists, and biased ligands designed to engage specific signaling pathways while minimizing off-target effects. Notably, the non-peptidergic OTR agonist LIT-001 has shown efficacy in preclinical models of pain and social dysfunction, illustrating the potential of next-generation compounds (Frantz et al., 2018; Hilfiger et al., 2020; Piotrowska et al., 2024).

### 10.1 Preclinical evidence

Chronic and acute treatment of healthy mice with intranasal OT or VP produce diverse effects in functional neuroimaging (Zhao et al., 2026). Intranasal OT acutely activates many cortical areas, and more persistently subcortical areas in the limbic system enriched with OTR, while intranasal VP persistently deactivates parietal cortex, thalamus and mesolimbic areas. These patterns are not reproduced by venous injections nor by analogs targeting selectively OTR, thus excluding the possibility of receptor crosstalk (Galbusera et al., 2017).

Experimental studies in multiple rodent models demonstrate that manipulation of OT/VP signaling can strongly influence social behavior, stress responsiveness, and reward processing. Intranasal or circuit-specific delivery of OT improves sociability, reduces anxiety-like behavior, and enhances social learning, whereas V1a/V1b antagonism attenuates stress-related and addiction-like phenotypes (Meziane et al., 2015; Penagarikano et al., 2015; Oztan et al., 2020). Importantly, therapeutic efficacy often depends on developmental timing, brain region, and behavioral context, supporting the view that OT and VP act through distributed circuit motifs rather than uniform neuromodulatory mechanisms. For example, early post-natal treatment with OT ameliorated social and cognitive behaviors beyond termination of treatment in adulthood (Grinevich et al., 2014), indicating long-term effects that similar treatment later in life failed to replicate. Intranasal VP administration early in life improved social behavior in a monkey model of autism (Talbot et al., 2024). It is believed that early exposure to OT/VP around birth particularly in animals with deficient levels can influence neural circuit development and long-term behavioral outcomes.

### 10.2 Clinical studies

Despite encouraging preclinical results, clinical trials have produced mixed outcomes. In healthy individuals, intranasal OT and VP alter activity across social, emotional, and reward-related networks, with measurable effects on attention, social cognition, and functional connectivity (Kawada et al., 2019; Liu et al., 2019; Zhuang et al., 2021; Nave et al., 2015). However, meta-analyses report modest or inconsistent behavioral benefits, particularly for OT administration in healthy populations (Yang et al., 2021). Besides, uncertainty was showed to make OT effects more anxiogenic (Grillon et al., 2013), arguing, again that OT is best understood as a context-dependent modulator of social stimuli processing. Overall, human imaging indicates that intranasal OT or VP both elicit significant brain-wide changes specifically in socially-relevant states, holding potential for modification strategies in diseases featuring OT/VP disruptions (Zink and Meyer-Lindenberg, 2012; Zhao et al., 2026).

Results in neuropsychiatric disorders have been similarly variable (Leng and Ludwig, 2016; Zhao et al., 2026), and meta-analyses reported no effects (Leppanen et al., 2017; Yang et al., 2021). For example, in PTSD patients, OT levels have been reported decreased in males peripheral milieu, while VP levels were normal (Frijling et al., 2015). OT injection generally attenuates amygdala reactivity (Koch et al., 2016) but the contrary was also reported (Frijling et al., 2016). In addition to changing brain activity, intranasal OT has been shown to prevent PTSD symptoms (Van Zuiden et al., 2017), but this effect may depend on the memory phase during which OT is administered (as shown in animal models; Campbell-Smith et al., 2015). It may also depend on the context of trauma, as an intranasal OT treatment that reduces symptoms in high-risk PTSD patients showed limited efficacy in the broader-trauma exposed population (Frijling, 2017; Van Zuiden et al., 2017). In patients with autism, meta-analyses reported no significant therapeutic value for OT in treating core symptoms (Sikich et al., 2021; Hu et al., 2023), despite evidence for altered functional connectivity within social brain networks (Alaerts et al., 2020, 2022; Bernaerts et al., 2020). Early-life administration may represent an exception, as studies in Prader–Willi syndrome infants with all types of genetic anomalies suggest benefits when treatment is initiated during critical developmental window (Viaux-Savelon et al., 2016; Tauber et al., 2017; Rice et al., 2018; Valette et al., 2025), whereas similar treatment later in life provided mixed results (Shalma et al., 2023; Petersson and Höybye, 2024). Long-term impact on psychiatric trajectories remains unknown due to the need for extended longitudinal studies (Petersson and Höybye, 2024). Intranasal VP administration triggers similar improvements in 6–12 years-old children with autism, a critical period of neurodevelopment that is extended for VP compared to OT (Parker et al., 2019). Other studies administered the V1a antagonist Balovaptan in young adults with autism eliciting similar benefits in adaptive behaviors (Bolognani et al., 2019; Kimi et al., 2024). However, larger follow-up studies in children and young adults with autism have generally failed to replicate the initial findings (Hollander et al., 2022; Jacob et al., 2022).

### 10.3 Emerging perspective

Genetic variation in OTR and V1a/V1b/V2 receptors, epigenetic signatures, and functional neuroimaging measures may help identify individuals most likely to benefit from targeted interventions (Salles et al., 2024). A central challenge is that systemic administration lacks circuit specificity. Because OT and VP can exert distinct, and sometimes opposing, effects across different brain regions, global receptor activation may engage competing network mechanisms that obscure therapeutic benefit. Future strategies will therefore require greater precision, including receptor-biased ligands, circuit-selective delivery approaches, and biomarker-guided patient stratification. Furthermore, context-dependency for the efficacy of OT/VP therapeutics has been validated and Prader-Willi syndrome in mouse models (Pantouli et al., 2024), and in patients with autism (Le et al., 2022). For example, parental care plus caregiver attention act as strong context when infants receive OT early in postnatal development. The robust activation of orbitofrontal cortices in this condition could be a proxy for enhanced emotional salience (Tauber et al., 2017). Pairing cognitive exposure therapy with OT could ameliorate PTSD outcomes (Sack et al., 2017; Flanagan et al., 2018; Sippel et al., 2024). Clinically, combination therapy could improve adherence to the psychotherapy for PTSD. Question remains whether the contextual enhancement effect of OT could be extended to electroconvulsive therapy or transcranial brain stimulation. Such studies with VP are currently lacking. Taken together, current evidence suggests that successful therapeutic manipulation of OT/VP systems will require moving beyond peptide replacement toward circuit-based approaches that account for receptor localization, developmental timing, and behavioral context. To this end, positive social interactions can increase endogenous OT release, potentially reinforcing affiliative behaviors and reducing anxiety and stress reactivity (Flanagan et al., 2018; Sippel et al., 2024). Context and patient characteristics must be taken into account before administering OT or VP therapeutics given their strong context and timing dependency. For example, OT/VP administration during memory consolidation, extinction learning, retrieval or reconsolidation might result in opposing clinical efficacy. It is thus recommended to control for the timing/context at exposure by combining the administration with psychotherapy or circuit-guided intervention (electroconvulsive or transcranial magnetic stimulation).

## 11 A hypothetical circuit framework for oxytocin-vasopressin interactions

### 11.1 Push-pull modulation, synergistic amplification and parallel pathway segregation

**Figure 5:**
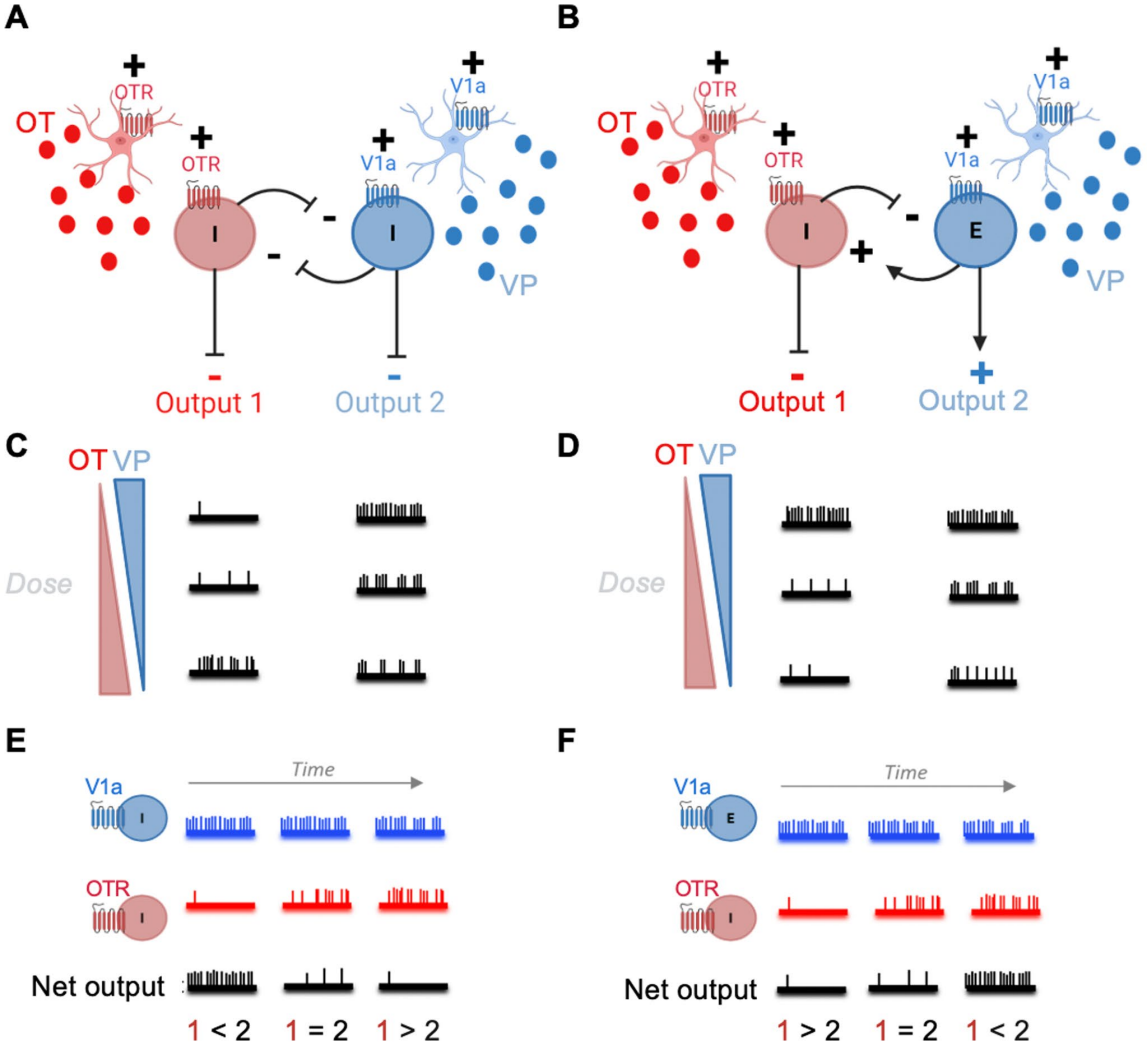
Hypothetical model. **(A)** Segregated actions of OT/VP in disinhibitory circuit motif and associated astrocytes. **(B)** Segregated actions of OT/VP in excitatory-inhibitory circuit motif and associated astrocytes. **(C)** Dose effect in motif A: When VP > OT, V1a cells increase firing, OTR cells decrease firing and feedback. When VP=OT, V1a cells activate, OTR cells shutdown but fail to feedback. When VP < OT, V1a cells silent, OTR cells shutdown and feedback. **(D)** Dose effect in motif B: When VP > OT, V1a cells activate, OTR cells activate backpropagating onto V1a cells. When VP=OT, V1a cells activate, OTR cells shutdown but fail to feedback. When VP < OT, V1a cells silent, OTR cells shutdown and back propagate inhibition on V1a cells. **(E)** Temporal dynamics in motif A: Short-term, OT + VP amplify inhibition acting as push pull mechanism in favor of output 2. Mid-term, VP dominates OT effects (transient) but outputs equalize because feedback inhibition fails. Long-term, VP effects sustain impact on output 1 more significantly than output 2. **(F)** Temporal dynamics in motif B: Short-term, OT + VP amplify output 1 and suppress output 2 by backpropagating inhibition onto V1a cells. Mid-term, VP dominates OT effects (transient) but outputs equalize because feedback inhibition fails. Long-term, VP effects sustain impact on output 2 by excitation. Created with Biorender.com.

The diverse and sometimes seemingly contradictory effects of OT and VP across brain systems may be interpreted within a working framework in which these peptides function as context-dependent modulators of neural circuit state (Figure 5). Rather than acting as simple pro-social or anxiety signals, OT and VP appear to dynamically regulate the balance between competing modes of network activity, including inhibition vs. excitation for impacting safety vs. threat, and parasympathetic vs. sympathetic dominance. Importantly, this framework should be viewed as a heuristic model intended to organize emerging circuit-level observations rather than as a universal principle established across all systems.

A recurring observation across several brain regions is that OT and VP signaling are embedded within conserved microcircuit motifs that shape information flow according to receptor localization, neuronal identity, and network topology. We propose three broad organizational circuit motifs: push-pull modulation, synergistic amplification, and parallel pathway segregation, through which peptidergic signaling may influence circuit computations (Besnard and Leroy, 2022). Although similar organizational principles emerge across multiple systems, the architecture and functional consequences of these motifs are likely region-, species-, sex-, and state-dependent.

Push-pull modulation has been described in structures including the central amygdala (CeA), bed nucleus of the stria terminalis (BNST), hippocampus, and lateral septum. In several of these circuits, VP increases excitatory drive or output activity (the “push”) whereas OT preferentially recruits inhibitory gating mechanisms (the “pull”). However, these effects are not universal and depend strongly on receptor distribution, developmental stage, synaptic polarity, and cellular context. For example, OT can either increase or suppress neuronal firing depending on whether OTRs are expressed in excitatory or inhibitory neurons, whether GABAergic signaling is depolarizing or hyperpolarizing, and whether signaling occurs through GABA_A_ or GABA_B_ mechanisms. Likewise, VP signaling through V1a and V1b receptors may engage distinct pre- and postsynaptic mechanisms. Consequently, behavioral output likely emerges from the relative balance of peptide release, receptor recruitment, and local circuit architecture rather than from fixed “OT vs. VP” functions.

Synergistic amplification may occur in circuits involved in sensory salience and social discrimination, including the olfactory bulb, lateral septum, and selected brainstem nuclei. In these systems, VP-mediated excitation and OT-mediated disinhibition can together increase signal-to-noise ratio and selectively enhance the transmission of behaviorally relevant inputs. Nevertheless, such cooperative interactions are unlikely to represent a universal rule. In some regions, OT and VP may instead exert opposing or temporally dissociated effects on network excitability. Thus, the functional role of these peptides should be interpreted within the specific topology and physiological state of each circuit.

Parallel pathway segregation has been described in sensorimotor and autonomic systems, where OT- and VP-sensitive pathways regulate distinct but coordinated physiological outputs. Such organization may allow behavioral state transitions to be coupled with appropriate endocrine, autonomic, and motivational responses. Importantly, these pathways are not fully independent, but are embedded within broader recurrent networks linking hypothalamic, limbic, and brainstem structures.

These motifs likely operate across hierarchical levels of brain organization. For example, OT/VP balance within amygdala-centered networks influences downstream autonomic pathways, while visceral feedback can in turn modulate limbic processing. Similarly, hippocampal regulation of contextual memory interacts with septal, hypothalamic, and amygdalar circuits to shape the conditions under which OT- or VP-dependent signaling predominates. Reciprocal connectivity between peptide-producing neurons and receptor-expressing target populations further suggests the existence of recurrent feedback loops capable of dynamically regulating network state (Huber et al., 2005; Tobin et al., 2010; Dromard et al., 2024). However, the precise architecture and causal significance of these circuit motifs remain incompletely resolved.

An important implication of this framework is that the functional outcome of OT/VP signaling cannot be inferred from peptide identity alone. The same peptide may produce distinct -and occasionally opposite- effects depending on where and when it is released, the receptors engaged, and the topology of the surrounding circuit. This context dependence may help explain the variability observed across experimental paradigms, species, and clinical studies, particularly those involving systemic or intranasal peptide administration.

Another emerging feature of OT/VP signaling is its operation across multiple temporal scales. OT often produces rapid modulation of synaptic transmission and neuronal excitability, whereas VP signaling has frequently been associated with longer-lasting intracellular and transcriptional effects linked to stress adaptation and synaptic plasticity. These temporally distinct dynamics may permit simultaneous short-term regulation of behavioral state and longer-term remodeling of circuit function.

### 11.2 Large scale functional states

From the alternative stable state theory point of view, like the homeodynamic perspective (Petitjean et al., 2025), brain circuits are not constrained to maintain a single homeostatic equilibrium but may transition between multiple stable operating regimes according to environmental demands, physiological needs, or pathological conditions. In this framework, OT and VP could be viewed as modulators of state transitions, acting on distributed feedback loops that regulate the balance between competing behavioral programs such as safety vs. threat, social engagement vs. withdrawal, or nociceptive facilitation vs. inhibition.

Such a view is consistent with the context-dependent and multi-scale actions of OT/VP signaling described above. Rather than restoring a predefined equilibrium, these neuropeptides may facilitate adaptive reorganization of neural networks, allowing the system to settle into alternative stable states that optimize survival under changing internal and external conditions. Conversely, maladaptive stabilization of these states may contribute to chronic pathological conditions, including persistent pain, stress-related disorders, and social dysfunction.

From a broader computational perspective, OT and VP may contribute to state-dependent gain control across distributed social, emotional, autonomic, and reward-related networks. Rather than encoding discrete behavioral functions, these peptides may regulate how neural circuits prioritize salience, integrate internal state with environmental context, and transition between competing behavioral programs. Future studies integrating electrophysiology, circuit tracing, molecular profiling, and systems-level imaging will be required to determine the extent to which these proposed motifs represent conserved organizational principles vs. region-specific implementations of peptidergic neuromodulation.

## 12 Limitations and future directions

### 12.1 Limitations

Major methodological and conceptual limitations continue to constrain interpretation of OT/VP research. Peripheral measurements of OT and VP remain highly variable and are strongly influenced by sample type, extraction methods, assay specificity, and peptide binding to plasma proteins, with poor correspondence between peripheral and central concentrations (Veening and Olivier, 2013; MacLean et al., 2017; Martins et al., 2020; Tabak et al., 2023). Similar challenges affect intranasal delivery studies, as the extent, timing, and mechanisms of brain penetration remain incompletely understood. Behavioral outcomes are further complicated by variability in dosage, timing, social context across experimental paradigms, and often a biased focus toward a specific behavior rather than a complete, too complicated, phenotyping.

Translation from animal models to humans also remains difficult. OT and VP systems exhibit pronounced species-, sex-, and context-dependent differences in receptor distribution and behavioral function (Insel, 2010; Albers, 2015; Dumais and Veenema, 2016). Circuit mechanisms identified in transgenic mouse models may therefore not generalize across species or disease states. In addition, receptor promiscuity and off-target pharmacology complicate interpretation of both experimental and clinical studies, underscoring the need for more selective ligands and rigorous electrophysiological validation. These difficulties might be integrated within future computational modeling and systematic reviews of the literature.

### 12.2 Future research directions

Major questions remain regarding how OT and VP achieve cell-type-, circuit-, and state-specific modulation across the brain. Although both peptides act on excitatory and inhibitory neurons, the precise identity, connectivity, and computational roles of receptor-expressing populations remain incompletely resolved. Emerging single-cell, spatial transcriptomic, and connectomic approaches will be essential to define these circuit architectures with higher precision.

Another critical challenge is to understand the spatiotemporal logic of OT/VP release. These neurons operate through both synaptic and volume transmission, often co-releasing glutamate alongside neuropeptides (Kawasaki et al., 2005; Hasan et al., 2019; Zhang et al., 2020; Otubo et al., 2021; Inada et al., 2022; Thirtamara Rajamani et al., 2024), thereby coupling fast excitation with slower neuromodulation. How activity patterns recruit distinct modes of co-transmission, receptor engagement, and long-range circuit coordination remains poorly understood. Receptor heterodimerization (Devost and Zingg, 2004; Amato et al., 2022; Borroto-Escuela et al., 2022), and peptide-transmitter interactions may further diversify signaling states and represent promising therapeutic targets (Busnelli et al., 2026).

Furthermore, the diversity of central nervous system cellular targets of neuropeptides adds a level of complexity in studying the intrication of OT and VP functions. While it is now established that astrocytes express neuropeptides receptors, among which OT, and play an important role in the OT functions, little is known about other glial cells, such as microglia and oligodendrocytes. Novel studies will be needed to enlighten their putative role in OT/VP regulatory effects on brain functions.

Future progress will require integrating molecular profiling, circuit electrophysiology, *in vivo* imaging, and computational neuroscience to construct a unified systems-level model of OT/VP neuromodulation of circuits that do not operate in isolation but are embedded within broader architectures involving dopamine, serotonin, CRF, somatostatin and neurokinin systems (Froemke and Young, 2021). How these interactions coordinate higher-order circuit-state transitions across social, reward, and stress networks remain largely overlooked.

OTR expression in dopaminergic neurons of the ventral tegmental area and dopamine-induced OT release from the PVN positions OT as feedforward regulator of salience, reinforcement, and motivational drive within social contexts (Baskerville and Douglas, 2010; Hung et al., 2017; Liu et al., 2020; Musardo et al., 2022; Sun et al., 2025; Matsui et al., 2026). OTR may heterodimerize with dopamine receptor 2 in regions like the striatum, nucleus accumbens and amygdala where it is thought to influence memory updating/reconsolidation with social stimuli (Hernández-Mondragón et al., 2023).

OT/VP signaling through CRF-related septal and hypothalamic circuits may amplify vigilance and stress responsivity (Albeck et al., 1997; Nair et al., 2005; Morisot et al., 2018; Winter and Jurek, 2019; Loewen et al., 2020; Winter et al., 2023). Reciprocally, CRF1 and CRF2 are expressed in OT and VP neurons in the SON and PVN (Arima and Aguilera, 2000). Elevated CRF1 coincides with lower OTR in amygdala and prefrontal cortex of a rat PTSD model, which symptoms could be improved by intranasal OT injections (Wang et al., 2019).

Serotonergic and somatostatinergic systems likely further tune these circuit motifs by shaping excitatory–inhibitory balance, neuropeptide release at precise sites of circuit motifs, behavioral flexibility, and affective state (Andari, 2021; Grieb et al., 2021; Borie et al., 2024; Dromard et al., 2024). Following acute stress, lower serotonin and dopamine levels in prefrontal cortex coincides with higher norepinephrine in prefrontal cortex and lower circulating cortiscosterone levels, higher sociability and anxiety, all of which reverted upon injections of the OTR antagonist L368899. This treatment did not however affect stress-induced changes in neurotransmitters in the amygdala (Wang et al., 2025). Such convergence suggests that OT/VP motifs are nested within larger neuromodulatory systems that regulate behavioral state allocation. Similar principles emerge in mesolimbic circuits where dopamine and substance P exert complementary or opposing actions depending on receptor localization and circuit topology (Johnson et al., 2012), reinforcing the concept that neuromodulators operate through distributed microcircuit logic rather than isolated transmitter-specific functions.

We propose that OT/VP signaling should be viewed as one layer of an integrated neuromodulatory code that filters signal output by switching between tonic-burst activity via precisely coordinated excitation and inhibition with consequences on stress responsiveness, reward valuation, and social salience.

## 13 Conclusion

It is time to move beyond viewing OT and VP as isolated “social peptides” and instead understand them modulators of as a unified circuit architecture governing state transitions across the social brain. Future therapies will probably not emerge from indiscriminate peptide administration, but are more likely to emerge from resolving how OTR-, V1a-, and V1b-defined microcircuits become functionally uncoupled in disease. Integrating electrophysiology, circuit imaging, and genetic stratification will be essential for identifying pathological network states and designing precision interventions. In this framework, OT/VP signaling provides a mechanistic bridge between synaptic computation and complex behavior, offering a new foundation for translational psychiatry. Future treatment with OT/VP therapeutics will be embedded within mechanistically-informed behavioral paradigms.

## Glossary

ASD: autism spectrum disorders
AON: anterior olfactory nuclei
BLA: basolateral amygdala
BNSTdl: dorsolateral bed nucleus of the stria terminalis
CA1-3: cornus ammonis 1–3
CAMKII: calmodulin kinase II
CCK: cholecystokinin
CCKR: CCK receptor
CeA: central amygdala
CeL: central lateral amygdala
CeM: central medial amygdala
CREB: *cyclic AMP response element-binding protein*
CRF: corticotropin-releasing factor
CRF2: CRF receptor 2
DG: dentate gyrus
DLPFC: dorsolateral prefrontal cortex
DMN: dorsal motor nucleus of the vagus nerve
ERK: extracellularly regulated kinase
EPSP: excitatory postsynaptic potential
GABA: gamma-aminobutyric acid
GABA-A: GABA receptor type A
GABA-B: GABA receptor type B
GCG: glucagon
GLP1R: GCG-related peptide 1 receptor
GFAP: Glial Fibrillary Acidic Protein
Gly: glycine
GPR83: G-protein-coupled-receptor-83
GC: granule cells
HPA: hypothalamic–pituitary–adrenal
HPN: hypoglossal nucleus
i.c.v.: intracerebroventricular
KCC2: potassium chloride cotransporter 2
LS: lateral septum
LTP: long-term potentiation
MOB: main olfactory bulb
MeA: median amygdala
MC: mitral cells
NAc: nucleus accumbens
NDNF: neuron-derived neurotrophic factor
NK1: neurokinin receptor 1
NTS: nucleus of the solitary tract
OE: olfactory epithelium
OT: oxytocin
OTR: oxytocin receptor
PAG: periaqueductal gray
PBN: parabrachial nucleus
PKCd: protein kinase C delta
PrBo: pre-Botzinger complex
OSN: olfactory sensory neurons
PTSD: post-traumatic stress disorder
PV: parvalbumin
PVN: paraventricular nucleus
Rock: Rho-associated coiled-coil kinase
RVLM: rostral ventrolateral medulla
SON: supraoptic nucleus
SCN: suprachiasmatic nucleus
SST: somatostatin
SUM: supra mammillary nucleus
TGT-b: transforming growth factor beta
TH: tyrosine hydroxylase
TRPV1: Transient Receptor Potential Cation Channel Subfamily V Member 1
V1a: VP receptor 1a
V1b: VP receptor 1b
V2: VP receptor 2
VP: vasopressin
VTA: ventral tegmental area.

## References

[ref1] Alaerts K. Bernaerts S. Prinsen J. Dillen C. Steyaert J. Wenderoth N. (2020). Oxytocin induces long-lasting adaptations within amygdala circuitry in autism: a treatment-mechanism study with randomized placebo-controlled design. Neuropsychopharmacol. 45, 1141–1149. doi: 10.1038/s41386-020-0653-8, 32161366 PMC7234999

[ref2] Alaerts K. Bernaerts S. Wenderoth N. (2022). Effects of single- and multiple-dose oxytocin treatment on amygdala low-frequency BOLD fluctuations and BOLD spectral dynamics in autism. Transl. Psychiatry 12:393. doi: 10.1038/s41398-022-02158-8, 36127337 PMC9489696

[ref3] Albeck D. S. McKittrick C. R. Blanchard D. C. Blanchard R. J. Nikulina J. McEwen B. S. (1997). Chronic social stress alters levels of corticotropin-releasing factor and arginine vasopressin mRNA in rat brain. J. Neurosci. 17, 4895–4903. 9169547 10.1523/JNEUROSCI.17-12-04895.1997 PMC6573358

[ref4] Albers H. E. (2015). Species, sex and individual differences in the vasotocin/vasopressin system: relationship to neurochemical signaling in the social behavior neural network. Front. Neuroendocrinol. 36, 49–71. doi: 10.1016/j.yfrne.2014.07.001, 25102443 PMC4317378

[ref5] Amato S. Averna M. Guidolin D. Pedrazzi M. Pelassa S. Capraro M. (2022). Heterodimer of A2A and oxytocin receptors regulating glutamate release in adult striatal astrocytes. IJMS 23:2326. doi: 10.3390/ijms23042326, 35216441 PMC8879615

[ref6] Andari E. (2021). New avenues for serotonin and oxytocin as a potential combination drug treatment for neuropsychiatric disorders. Biol. Psychiatry: Cognit. Neurosci. Neuroimaging 6, 1038–1039. doi: 10.1016/j.bpsc.2021.08.002, 34753609

[ref7] Apter-Levy Y. Feldman M. Vakart A. Ebstein R. P. Feldman R. (2013). Impact of maternal depression across the first 6 years of life on the child’s mental health, social engagement, and empathy: the moderating role of oxytocin. Am. J. Psychiatry 170, 1161–1168. doi: 10.1176/appi.ajp.2013.12121597, 23846912

[ref8] Arakawa H. Arakawa K. Deak T. (2010). Oxytocin and vasopressin in the medial amygdala differentially modulate approach and avoidance behavior toward illness-related social odor. Neuroscience 171, 1141–1151. doi: 10.1016/j.neuroscience.2010.10.013, 20933576

[ref9] Arima H. Aguilera G. (2000). Vasopressin and oxytocin neurones of hypothalamic supraoptic and paraventricular nuclei co-express mRNA for Type-1 andType-2 corticotropin-releasing hormone receptors. J. Neuroendocrinol. 12, 833–842. doi: 10.1046/j.1365-2826.2000.00528.x, 10971808

[ref10] Arnoux I. Garnero M. Moulard J. Rollenhagen A. Meinung C. (2026). Astrocytes Control Oxytocin-based Maternal Behavior via Connexin. Biorxiv, 30. doi: 10.64898/2026.01.09.698569

[ref11] Bakalkin G. Watanabe H. Kobikov Y. Li G. Nosova O. Rich K. (2026). The hypothalamic vasopressin circuit drives lateralized endocrine signaling. Neurobiol. Dis. 221:107340. doi: 10.1016/j.nbd.2026.107340, 41780699

[ref12] Baskerville T. A. Douglas A. J. (2010). Dopamine and oxytocin interactions underlying Behaviors: potential contributions to Behavioral disorders. CNS Neurosci. Ther. 16, e92–e123. doi: 10.1111/j.1755-5949.2010.00154.x, 20557568 PMC6493805

[ref13] Baudon A. Grelot V. Wang K.-Y. Althammer F. Denis C. Riché-Piotaix P. . (2025). Stress induces oxytocin-Gαi-dependent remodeling of astrocytes to shape neuronal response in the amygdala. Nat. Commun. 17:1364. doi: 10.1038/s41467-025-68114-4, 41462022 PMC12877189

[ref14] Bernaerts S. Boets B. Steyaert J. Wenderoth N. Alaerts K. (2020). Oxytocin treatment attenuates amygdala activity in autism: a treatment-mechanism study with long-term follow-up. Transl. Psychiatry 10:383. doi: 10.1038/s41398-020-01069-w, 33159033 PMC7648620

[ref15] Besnard A. Leroy F. (2022). Top-down regulation of motivated behaviors via lateral septum sub-circuits. Mol. Psychiatry 27, 3119–3128. doi: 10.1038/s41380-022-01599-3, 35581296 PMC7613864

[ref16] Bichet D. Bouvier M. Chini B. Gimpl G. Guillon G. Kimura T. . (2019). Vasopressin and oxytocin receptors (version 2019.4) in the IUPHAR/BPS guide to pharmacology database. IUPHAR/BPS Guide to Pharmacol. CITE 2019. doi: 10.2218/gtopdb/F66/2019.4

[ref17] Bohic M. Salamone P. C. Zuo W. Negm A. Fulton S. L. Du S. . (2026). Oxytocin modulation of spinal circuits drives therapeutic benefits of massage. BiorXiv. doi: 10.64898/2026.01.11.698886, 41648209 PMC12871349

[ref18] Bohus B. Urban I. Vanwimersmagreidanus T. Dewied D. (1978). Opposite effects of oxytocin and vasopressin on avoidance behaviour and hippocampal theta rhythm in the rat. Neuropharmacology 17, 239–247. 652136 10.1016/0028-3908(78)90107-7

[ref19] Boi L. Menon R. Denis C. Wang K.-Y. Petitjean H., (2026). Sex-Dependent Involvement of Lateral Septum Astrocytes in Social Fear: Role of Oxytocin Receptor Signaling. Biorxiv. doi: 10.64898/2026.02.01.703181

[ref20] Bolognani F. Del Valle Rubido M. Squassante L. Wandel C. Derks M. Murtagh L. . (2019). A phase 2 clinical trial of a vasopressin V1a receptor antagonist shows improved adaptive behaviors in men with autism spectrum disorder. Sci. Transl. Med. 11:eaat7838. doi: 10.1126/scitranslmed.aat7838, 31043521

[ref21] Bolte K. Wealing J. Revill A. (2023). Arginine vasopressin potentiates inspiratory bursting in hypoglossal motoneurons of neonatal mice. Respir. Physiol. Neurobiol. 314:104087. doi: 10.1016/j.resp.2023.104087, 37269889 PMC10443434

[ref22] Borie A. M. Agezo S. Lunsford P. Boender A. J. Guo J. D. Zhu H. . (2022). Social experience alters oxytocinergic modulation in the nucleus accumbens of female prairie voles. Curr. Biol. 32, 1026–1037e4. doi: 10.1016/j.cub.2022.01.014, 35108521 PMC8930613

[ref23] Borie A. Chakraborty P. Fontanaud P. Andre E. M. Francois A. Colson P. . (2024). Neuropeptide therapeutics to repress lateral septum neurons that disable sociability in an autism mouse model. Cell Rep. Med. 5:101781. doi: 10.1016/j.xcrm.2024.101781, 39423809 PMC11604546

[ref24] Borie A. M. Dromard Y. Guillon G. Olma A. Manning M. Muscatelli F. (2021a). Correction of vasopressin deficit in the lateral septum ameliorates social deficits of mouse autism model. J. Clin. Invest. 131:e144450. doi: 10.1172/JCI144450, 33232306 PMC7810497

[ref25] Borie A. M. Theofanopoulou C. Andari E. (2021b). “The promiscuity of the oxytocin–vasopressin systems and their involvement in autism spectrum disorder,” in Handbook of Clinical Neurology, (Amsterdam, Netherlands: Elsevier), 121–140. 10.1016/B978-0-12-819973-2.00009-5 PMC8637151 34266588

[ref26] Borroto-Escuela D. O. Cuesta-Marti C. Lopez-Salas A. Chruścicka-Smaga B. Crespo-Ramírez M. Tesoro-Cruz E. (2022). The oxytocin receptor represents a key hub in the GPCR heteroreceptor network: potential relevance for brain and behavior. Front. Mol. Neurosci. 15:1055344. doi: 10.3389/fnmol.2022.1055344, 36618821 PMC9812438

[ref27] Bortolozzo-Gleich M. H. Bouisset G. Geng L. Pino A. R. Nomura Y. Han S. (2025). Impaired vasopressin neuromodulation of the lateral septum leads to social behavior deficits in Shank3B+/− male mice. Nat. Commun. 16:6783. doi: 10.1038/s41467-025-61994-6, 40702006 PMC12287534

[ref28] Bowen M. T. Neumann I. D. (2017). Rebalancing the addicted brain: oxytocin interference with the neural substrates of addiction. Trends Neurosci. 40, 691–708. doi: 10.1016/j.tins.2017.10.003, 29128108

[ref29] Brierley D. I. Holt M. K. Singh A. De Araujo A. McDougle M. Vergara M. . (2021). Central and peripheral GLP-1 systems independently suppress eating. Nat. Metab. 3, 258–273. doi: 10.1038/s42255-021-00344-4, 33589843 PMC7116821

[ref30] Brinton R. D. Yamazaki R. Gonzalez C. M. O’Neill K. Schreiber S. S. (1998). Vasopressin-induction of the immediate early gene, NGFI-A, in cultured hippocampal glial cells. Brain Res. Mol. Brain Res. 57, 73–85. 9630527 10.1016/s0169-328x(98)00069-2

[ref31] Brown C. H. Bains J. S. Ludwig M. Stern J. E. (2013). Physiological regulation of magnocellular neurosecretory cell activity: integration of intrinsic, local and afferent mechanisms. J. Neuroendocrinol. 25, 678–710. doi: 10.1111/jne.12051, 23701531 PMC3852704

[ref32] Buron J. Linossier A. Gestreau C. Schaller F. Tyzio R. Felix M.-S. (2025). Oxytocin modulates respiratory heart rate variability through a hypothalamus–brainstem–heart neuronal pathway. Nat. Neurosci. 28, 2247–2261. doi: 10.1038/s41593-025-02074-2, 41116116 PMC12586189

[ref33] Busnelli M. Chini B. (2017). “Molecular basis of oxytocin receptor signalling in the brain: what we know and what we need to know,” in Behavioral Pharmacology of Neuropeptides: Oxytocin, eds. Hurlemann R. Grinevich V. (Cham: Springer International Publishing), 3–29. 10.1007/7854_2017_6 28812263

[ref34] Busnelli M. Gori A. Chini B. (2026). Bivalent ligands targeting oxytocin receptor homodimers selectively activate distinct G protein and β-arrestin pathways. Biochem. Pharmacol. 247:117814. doi: 10.1016/j.bcp.2026.117814, 41707738

[ref35] Busnelli M. Saulière A. Manning M. Bouvier M. Galés C. Chini B. (2012). Functional selective oxytocin-derived agonists discriminate between individual G protein family subtypes. J. Biol. Chem. 287, 3617–3629. doi: 10.1074/jbc.M111.277178, 22069312 PMC3281696

[ref36] Caffé A. R. Van Ryen P. C. Van Der Woude T. P. Van Leeuwen F. W. (1989). Vasopressin and oxytocin systems in the brain and upper spinal cord of Macaca fascicularis. J. Comp. Neurol. 287, 302–325. 2778107 10.1002/cne.902870304

[ref37] Campbell-Smith E. J. Holmes N. M. Lingawi N. W. Panayi M. C. Westbrook R. F. (2015). Oxytocin signaling in basolateral and central amygdala nuclei differentially regulates the acquisition, expression, and extinction of context-conditioned fear in rats. Learn. Mem. 22, 247–257. doi: 10.1101/lm.036962.114, 25878137 PMC4408769

[ref38] Caughey S. D. Klampfl S. M. Bishop V. R. Pfoertsch J. Neumann I. D. Bosch O. J. (2011). Changes in the intensity of maternal aggression and central oxytocin and vasopressin V1a receptors across the peripartum period in the rat. J. Neuroendocrinol. 23, 1113–1124. doi: 10.1111/j.1365-2826.2011.02224.x, 21929717

[ref39] Cechetto D. F. Saper C. B. (1988). Neurochemical organization of the hypothalamic projection to the spinal cord in the rat. J. Comp. Neurol. 272, 579–604. 2901438 10.1002/cne.902720410

[ref40] Chaki S. (2021). Vasopressin V1B receptor antagonists as potential antidepressants. Int. J. Neuropsychopharmacol. 24, 450–463. doi: 10.1093/ijnp/pyab013, 33733667 PMC8278797

[ref41] Chakraborty P. Lamat H. André E. M. Fontanaud P. Jeanneteau F. (2024). Acquiring social safety engages oxytocin neurons in the supraoptic nucleus: role of Magel2 deficiency. Neuroendocrinology 115, 1–16. doi: 10.1159/000538437, 38574475

[ref42] Chen S. He L. Huang A. J. Y. Boehringer R. Robert V. Wintzer M. E. . (2020). A hypothalamic novelty signal modulates hippocampal memory. Nature 586, 270–274. doi: 10.1038/s41586-020-2771-1, 32999460

[ref43] Chepkova A. N. French P. De Wied D. Ontskul A. H. Ramakers G. M. J. Skrebitski V. G. . (1995). Long-lasting enhancement of synaptic excitability of CA1/subiculum neurons of the rat ventral hippocampus by vasopressin and vasopressin(4–8). Brain Res. 701, 255–266. 8925289 10.1016/0006-8993(95)01006-7

[ref44] Chevaleyre V. Siegelbaum S. A. (2010). Strong CA2 pyramidal neuron synapses define a powerful Disynaptic Cortico-hippocampal loop. Neuron 66, 560–572. doi: 10.1016/j.neuron.2010.04.013, 20510860 PMC2905041

[ref45] Cilz N. I. Cymerblit-Sabba A. Young W. S. (2019). Oxytocin and vasopressin in the rodent hippocampus. Genes Brain Behav. 18:e12535. doi: 10.1111/gbb.12535, 30378258

[ref46] Clarke L. Gesundheit N. Sherr E. H. Hardan A. Y. Parker K. J. (2024). Vasopressin deficiency: a hypothesized driver of both social impairment and fluid imbalance in autism spectrum disorder. Mol. Psychiatry 29, 2568–2570. doi: 10.1038/s41380-024-02497-6, 38454082 PMC11380037

[ref47] Condés-Lara M. Rojas-Piloni G. Martínez-Lorenzana G. Rodríguez-Jiménez J. López Hidalgo M. Freund-Mercier M. J. (2006). Paraventricular hypothalamic influences on spinal nociceptive processing. Brain Res. 1081, 126–137. doi: 10.1016/j.brainres.2006.01.050, 16497280

[ref48] Corbani M. Marir R. Trueba M. Chafai M. Vincent A. Borie A. M. (2018). Neuroanatomical distribution and function of the vasopressin V1B receptor in the rat brain deciphered using specific fluorescent ligands. Gen. Comp. Endocrinol. 258, 15–32. doi: 10.1016/j.ygcen.2017.10.011, 29155265

[ref49] Cruz M. T. Dezfuli G. Murphy E. C. Vicini S. Sahibzada N. Gillis R. A. (2019). GABAB receptor Signaling in the dorsal motor nucleus of the Vagus stimulates gastric motility via a cholinergic pathway. Front. Neurosci. 13:967. doi: 10.3389/fnins.2019.00967, 31572117 PMC6751316

[ref50] Csikota P. Fodor A. Balázsfi D. Pintér O. Mizukami H. Weger S. (2016). Vasopressinergic control of stress-related behavior: studies in Brattleboro rats. Stress 19, 349–361. doi: 10.1080/10253890.2016.1183117, 27187740

[ref51] Cymerblit-Sabba A. Walsh C. Duan K.-Z. Song J. Holmes O. Young W. S. (2023). Simultaneous Knockouts of the Oxytocin and Vasopressin 1b Receptors in Hippocampal CA2 Impair social memory. BioRxiv [Preprint]. 2023.01.30.526271. doi: 10.1101/2023.01.30.526271

[ref52] Devost D. Zingg H. H. (2004). Homo- and hetero-dimeric complex formations of the human oxytocin receptor. J. Neuroendocrinol. 16, 372–377. doi: 10.1111/j.0953-8194.2004.01188.x, 15089977

[ref53] Di Scala-Guenot D. Mouginot D. Strosser M. (1994). Increase of intracellular calcium induced by oxytocin in hypothalamic cultured astrocytes. Glia 11, 269–276. 7960031 10.1002/glia.440110308

[ref54] Dluzen D. E. Muraoka S. Engelmann M. Landgraf R. (1998). The effects of infusion of arginine vasopressin, oxytocin, or their antagonists into the olfactory bulb upon social recognition responses in male rats. Peptides 19, 999–1005. 9700747 10.1016/s0196-9781(98)00047-3

[ref55] Dromard Y. Borie A. M. Chakraborty P. Muscatelli F. Guillon G. Desarménien M. G. (2024). Disengagement of somatostatin neurons from lateral septum circuitry by oxytocin and vasopressin restores social fear extinction and suppresses aggression outbursts in a Prader-Willi syndrome model. Biol. Psychiatry 95, 785–799. doi: 10.1016/j.biopsych.2023.10.016, 38952926 PMC11216544

[ref56] Dumais K. M. Veenema A. H. (2016). Vasopressin and oxytocin receptor systems in the brain: sex differences and sex-specific regulation of social behavior. Front. Neuroendocrinol. 40, 1–23. doi: 10.1016/j.yfrne.2015.04.003, 25951955 PMC4633405

[ref57] Duque-Wilckens N. Steinman M. Q. Busnelli M. Chini B. Yokoyama S. Pham M. (2018). Oxytocin receptors in the anteromedial bed nucleus of the stria terminalis promote stress-induced social avoidance in female California mice. Biol. Psychiatry 83, 203–213. doi: 10.1016/j.biopsych.2017.08.024, 29066224 PMC5743604

[ref58] Edwards S. Guerrero M. Ghoneim O. M. Roberts E. Koob G. F. (2012). Evidence that vasopressin V_1b_ receptors mediate the transition to excessive drinking in ethanol-dependent rats. Addict. Biol. 17, 76–85. doi: 10.1111/j.1369-1600.2010.00291.x, 21309953 PMC3178679

[ref59] Eliava M. Melchior M. Knobloch-Bollmann H. S. Wahis J. da Silva Gouveia M. Tang Y. (2016). A new population of parvocellular oxytocin neurons controlling magnocellular neuron activity and inflammatory pain processing. Neuron 89, 1291–1304. doi: 10.1016/j.neuron.2016.01.041, 26948889 PMC5679079

[ref60] Elsaafien K. Kirchner M. K. Baumer-Harrison C. Tan Y. Johnson D. N. Vincent C. J. (2026). Oxytocin and vasopressin cross talk within the brain increases blood pressure. Circ. Res. 138:e327322. doi: 10.1161/CIRCRESAHA.125.327322, 41616097 PMC12904227

[ref61] Esmaeilou Y. Tamaddonfard E. Erfanparast A. Soltanalinejad-Taghiabad F. (2022). Behavioral and receptor expression studies on the primary somatosensory cortex and anterior cingulate cortex oxytocin involvement in modulation of sensory and affective dimensions of neuropathic pain induced by partial sciatic nerve ligation in rats. Physiol. Behav. 251:113818. doi: 10.1016/j.physbeh.2022.113818, 35443199

[ref62] Fan F. Cao Y. He Z.-Q. Yang F. Chen Y. Chen A.-Q. . (2026). Oxytocin relieves visceral hypersensitivity through GABAB1-TRPV1 in rats with irritable bowel syndrome. Sheng Li Xue Bao 78, 173–181. doi: 10.1111/adb.13025 41777139

[ref63] Fan X. Shi G. He X. Li X. Wan Y. Jian L. (2021). Oxytocin prevents cue-induced reinstatement of oxycodone seeking: involvement of DNA methylation in the hippocampus. Addict. Biol. 26:e13025. doi: 10.1111/adb.13025, 33609013

[ref64] Fang L.-Y. Quan R.-D. Kaba H. (2008). Oxytocin facilitates the induction of long-term potentiation in the accessory olfactory bulb. Neurosci. Lett. 438, 133–137. doi: 10.1016/j.neulet.2007.12.070, 18468792

[ref65] Feldman R. Vengrober A. Ebstein R. P. (2014). Affiliation buffers stress: cumulative genetic risk in oxytocin–vasopressin genes combines with early caregiving to predict PTSD in war-exposed young children. Transl. Psychiatry 4, e370–e370. doi: 10.1038/tp.2014.6, 24618689 PMC3966045

[ref66] Ferguson J. N. Young L. J. Hearn E. F. Matzuk M. M. Insel T. R. Winslow J. T. (2000). Social amnesia in mice lacking the oxytocin gene. Nat. Genet. 25, 284–288. doi: 10.1038/77040, 10888874

[ref67] Flanagan J. C. Sippel L. M. Wahlquist A. Moran-Santa Maria M. M. Back S. E. (2018). Augmenting prolonged exposure therapy for PTSD with intranasal oxytocin: a randomized, placebo-controlled pilot trial. J. Psychiatr. Res. 98, 64–69. doi: 10.1016/j.jpsychires.2017.12.014, 29294429 PMC5800951

[ref68] Flanigan M. E. Kash T. L. (2022). Coordination of social behaviors by the bed nucleus of the stria terminalis. Eur. J. Neurosci. 55, 2404–2420. doi: 10.1111/ejn.14991, 33006806 PMC9906816

[ref69] Francesconi W. Olivera-Pasilio V. Berton F. Olson S. L. Chudoba R. Monroy L. M. (2025). Vasopressin and oxytocin excite BNST neurons via oxytocin receptors, which reduce anxious arousal. Cell Rep. 44:115768. doi: 10.1016/j.celrep.2025.115768, 40471787 PMC12294564

[ref70] François M. Vranich K. L. Delgado I. C. Lafond A. Lopatinsky N. R. Davis T. (2025). Amygdala AVPR1A mediates susceptibility to chronic social isolation in female mice. Nat. Commun. 16:9740. doi: 10.1038/s41467-025-64742-y, 41188217 PMC12586453

[ref71] Frantz M.-C. Pellissier L. P. Pflimlin E. Loison S. Gandía J. Marsol C. (2018). LIT-001, the first nonpeptide oxytocin receptor agonist that improves social interaction in a mouse model of autism. J. Med. Chem. 61, 8670–8692. doi: 10.1021/acs.jmedchem.8b00697, 30199637

[ref72] Frijling J. L. (2017). Preventing PTSD with oxytocin: effects of oxytocin administration on fear neurocircuitry and PTSD symptom development in recently trauma-exposed individuals. Eur. J. Psychotraumatol. 8:1302652. doi: 10.1080/20008198.2017.1302652, 28451068 PMC5400019

[ref73] Frijling J. L. Van Zuiden M. Koch S. B. J. Nawijn L. Veltman D. J. Olff M. (2016). Effects of intranasal oxytocin on amygdala reactivity to emotional faces in recently trauma-exposed individuals. Soc. Cogn. Affect. Neurosci. 11, 327–336. doi: 10.1093/scan/nsv116, 26382634 PMC4733344

[ref74] Frijling J. L. Van Zuiden M. Nawijn L. Koch S. B. J. Neumann I. D. Veltman D. J. . (2015). Salivary oxytocin and vasopressin levels in police officers with and without post-traumatic stress disorder. J. Neuroendocrinol. 27, 743–751. doi: 10.1111/jne.12300, 26184739

[ref75] Froemke R. C. Young L. J. (2021). Oxytocin, neural plasticity, and social behavior. Annu. Rev. Neurosci. 44, 359–381. doi: 10.1146/annurev-neuro-102320-102847, 33823654 PMC8604207

[ref76] Galbusera A. De Felice A. Girardi S. Bassetto G. Maschietto M. Nishimori K. . (2017). Intranasal oxytocin and vasopressin modulate divergent brainwide functional substrates. Neuropsychopharmacology 42, 1420–1434. doi: 10.1038/npp.2016.283, 27995932 PMC5436116

[ref77] García-Boll E. Martínez-Lorenzana G. Condés-Lara M. González-Hernández A. (2018). Oxytocin inhibits the rat medullary dorsal horn Sp5c/C1 nociceptive transmission through OT but not V 1A receptors. Neuropharmacology 129, 109–117. doi: 10.1016/j.neuropharm.2017.11.031, 29169960

[ref78] Gonzalez-Hernandez A. Charlet A. (2018). Oxytocin, GABA, and TRPV1, the analgesic triad? Front. Mol. Neurosci. 11:398. doi: 10.3389/fnmol.2018.00398, 30555298 PMC6282058

[ref79] Gravati M. Busnelli M. Bulgheroni E. Reversi A. Spaiardi P. Parenti M. (2010). Dual modulation of inward rectifier potassium currents in olfactory neuronal cells by promiscuous G protein coupling of the oxytocin receptor. J. Neurochem. 114, 1424–1435. doi: 10.1111/j.1471-4159.2010.06861.x, 20557424

[ref80] Grieb Z. A. Ross A. P. McCann K. E. Lee S. Welch M. Gomez M. G. (2021). Sex-dependent effects of social status on the regulation of arginine-vasopressin (AVP) V1a, oxytocin (OT), and serotonin (5-HT) 1A receptor binding and aggression in Syrian hamsters (*Mesocricetus auratus*). Horm. Behav. 127:104878. doi: 10.1016/j.yhbeh.2020.104878, 33148500 PMC8889570

[ref81] Grillon C. Krimsky M. Charney D. R. Vytal K. Ernst M. Cornwell B. (2013). Oxytocin increases anxiety to unpredictable threat. Mol. Psychiatry 18, 958–960. doi: 10.1038/mp.2012.156, 23147382 PMC3930442

[ref82] Grinevich V. Desarmenien M. G. Chini B. Tauber M. Muscatelli F. (2014). Ontogenesis of oxytocin pathways in the mammalian brain: late maturation and psychosocial disorders. Front. Neuroanat. 8:164. doi: 10.3389/fnana.2014.00164, 25767437 PMC4341354

[ref83] Grinevich V. Knobloch-Bollmann H. S. Eliava M. Busnelli M. Chini B. (2016). Assembling the puzzle: pathways of oxytocin Signaling in the brain. Biol. Psychiatry 79, 155–164. doi: 10.1016/j.biopsych.2015.04.013, 26001309

[ref84] Grinevich V. Stoop R. (2018). Interplay between oxytocin and sensory Systems in the Orchestration of socio-emotional Behaviors. Neuron 99, 887–904. doi: 10.1016/j.neuron.2018.07.016, 30189208

[ref85] Gruber T. Lechner F. Murat C. Contreras R. E. Sanchez-Quant E. Miok V. (2023). High-calorie diets uncouple hypothalamic oxytocin neurons from a gut-to-brain satiation pathway via κ-opioid signaling. Cell Rep. 42:113305. doi: 10.1016/j.celrep.2023.113305, 37864798 PMC10636643

[ref86] Grund T. Tang Y. Benusiglio D. Althammer F. Probst S. Oppenlander L. . (2019). Chemogenetic activation of oxytocin neurons: temporal dynamics, hormonal release, and behavioral consequences. Psychoneuroendocrinology 106, 77–84. doi: 10.1016/j.psyneuen.2019.03.019, 30954921

[ref87] Gu N. Makashova O. Laporte C. Chen C. Q. Li B. Chevillard P.-M. (2025). Microglia regulate neuronal activity via structural remodeling of astrocytes. Neuron 113, 3408–3423.e5. doi: 10.1016/j.neuron.2025.07.024, 40834861

[ref88] Gupta P. R. Prabhavalkar K. (2021). Combination therapy with neuropeptides for the treatment of anxiety disorder. Neuropeptides 86:102127. doi: 10.1016/j.npep.2021.102127, 33607407

[ref89] Hallbeck M. Larhammar D. Blomqvist A. (2001). Neuropeptide expression in rat paraventricular hypothalamic neurons that project to the spinal cord. J. Comp. Neurol. 433, 222–238. doi: 10.1002/cne.1137, 11283961

[ref90] Harrison T. Tao A. S. Vo P. Kim S. Kim S. (2026). Trajectories of changes in oxytocin and vasopressin before, during, and after mother-infant interaction: a descriptive study of mothers and infants affected by postpartum depression. Front. Psychol. 16:1636616. doi: 10.3389/fpsyt.2025.1636616, 41562003 PMC12813420

[ref91] Hartswick D. Zaw A. Schappaugh N. Friesen C. N. De Vries G. J. Petrulis A. (2025). Connectivity and Phenotype of Vasopressin 1a Receptor cells in the lateral septum. BioRxiv [Preprint]. 2025.10.26.683946. doi: 10.1101/2025.10.26.683946

[ref92] Hasan M. T. Althammer F. Silva Da Gouveia M. Goyon S. Eliava M. Lefevre A. . (2019). A fear memory engram and its plasticity in the hypothalamic oxytocin system. Neuron 103, 133–146.e8. doi: 10.1016/j.neuron.2019.04.029, 31104950

[ref93] Hatton G. I. Bicknell R. J. Hoyland J. Bunting R. Mason W. T. (1992). Arginine vasopressin mobilises intracellular calcium via V1-receptor activation in astrocytes (pituicytes) cultured from adult rat neural lobes. Brain Res. 588, 75–83. 1393572 10.1016/0006-8993(92)91346-g

[ref94] Hatton G. I. Perlmutter L. S. Salm A. K. Tweedle C. D. (1984). Dynamic neuronal-glial interactions in hypothalamus and pituitary: implications for control of hormone synthesis and release. Peptides 5, 121–138. 6384946 10.1016/0196-9781(84)90271-7

[ref95] Hernández-Mondragón J. C. Hernández-Hernández D. A. Crespo-Ramírez M. Prospero-García O. Rocha-Arrieta L. Fuxe K. . (2023). Evidence for the existence of facilitatory interactions between the dopamine D2 receptor and the oxytocin receptor in the amygdala of the rat. Relevance for anxiolytic actions. Front. Pharmacol. 14:1251922. doi: 10.3389/fphar.2023.1251922, 37900160 PMC10603234

[ref96] Hilfiger L. Zhao Q. Kerspern D. Inquimbert P. Andry V. Goumon Y. (2020). A nonpeptide oxytocin receptor agonist for a durable relief of inflammatory pain. Sci. Rep. 10:3017. doi: 10.1038/s41598-020-59929-w, 32080303 PMC7033278

[ref97] Hollander E. Jacob S. Jou R. McNamara N. Sikich L. Tobe R. (2022). Balovaptan vs placebo for social communication in childhood autism Spectrum disorder: a randomized clinical trial. JAMA Psychiatry 79, 760–769. doi: 10.1001/jamapsychiatry.2022.1717, 35793101 PMC9260643

[ref98] Horn A. J. Cole S. Nazarloo H. P. Nazarloo P. Davis J. M. Carrier D. (2024). Severe PTSD is marked by reduced oxytocin and elevated vasopressin. Comprehensive Psychoneuroendocrinol. 19:100236. doi: 10.1016/j.cpnec.2024.100236, 38764609 PMC11101686

[ref99] Hu L. Du X. Jiang Z. Song C. Liu D. (2023). Oxytocin treatment for core symptoms in children with autism spectrum disorder: a systematic review and meta-analysis. Eur. J. Clin. Pharmacol. 79, 1357–1363. doi: 10.1007/s00228-023-03545-w, 37540265

[ref100] Huang T. Guan F. Licinio J. Wong M. L. Yang Y. (2021). Activation of septal OXTr neurons induces anxiety- but not depressive-like behaviors. Mol. Psychiatry 26, 7270–7279. doi: 10.1038/s41380-021-01283-y, 34489531 PMC8873014

[ref101] Huber D. Veinante P. Stoop R. (2005). Vasopressin and oxytocin excite distinct neuronal populations in the central amygdala. Science 308, 245–248. doi: 10.1126/science.1105636, 15821089

[ref102] Hung L. W. Neuner S. Polepalli J. S. Beier K. T. Wright M. Walsh J. J. (2017). Gating of social reward by oxytocin in the ventral tegmental area. Science 357, 1406–1411. doi: 10.1126/science.aan4994, 28963257 PMC6214365

[ref103] Inada K. Hagihara M. Tsujimoto K. Abe T. Konno A. Hirai H. (2022). Plasticity of neural connections underlying oxytocin-mediated parental behaviors of male mice. Neuron 110, 2009–2023.e5. doi: 10.1016/j.neuron.2022.03.033, 35443152

[ref104] Inada K. Hagihara M. Yaguchi K. Irie S. Inoue Y. U. Inoue T. (2025). Vasopressin-to-oxytocin receptor crosstalk in the preoptic area underlying parental behaviors in male mice. Nat. Commun. 16:10844. doi: 10.1038/s41467-025-66908-0, 41372215 PMC12696063

[ref105] Insel T. R. (2010). The challenge of translation in social neuroscience: a review of oxytocin, vasopressin, and affiliative behavior. Neuron 65, 768–779. doi: 10.1016/j.neuron.2010.03.005, 20346754 PMC2847497

[ref106] Iwasaki M. Lefevre A. Althammer F. Clauss Creusot E. Łąpieś O. Petitjean H. (2023). An analgesic pathway from parvocellular oxytocin neurons to the periaqueductal gray in rats. Nat. Commun. 14:1066. doi: 10.1038/s41467-023-36641-7, 36828816 PMC9958129

[ref107] Jacob S. Veenstra-VanderWeele J. Murphy D. McCracken J. Smith J. Sanders K. (2022). Efficacy and safety of balovaptan for socialisation and communication difficulties in autistic adults in North America and Europe: a phase 3, randomised, placebo-controlled trial. Lancet Psychiatry 9, 199–210. doi: 10.1016/S2215-0366(21)00429-6, 35151410

[ref108] Joëls M. Urban I. J. A. (1984). Arginine8-vasopressin enhances the responses of lateral septal neurons in the rat to excitatory amino acids and fimbria-fornix stimuli. Brain Res. 311, 201–209. 6149788 10.1016/0006-8993(84)90084-2

[ref109] John S. Jaeggi A. V. (2021). Oxytocin levels tend to be lower in autistic children: a meta-analysis of 31 studies. Autism 25, 2152–2161. doi: 10.1177/13623613211034375, 34308675

[ref110] Johnson M. D. Hyngstrom A. S. Manuel M. Heckman C. J. (2012). Push-pull control of motor output. J. Neurosci. 32, 4592–4599. doi: 10.1523/JNEUROSCI.4709-11.2012, 22457505 PMC3335194

[ref111] Juif P.-E. Poisbeau P. (2013). Neurohormonal effects of oxytocin and vasopressin receptor agonists on spinal pain processing in male rats. Pain 154, 1449–1456. doi: 10.1016/j.pain.2013.05.003, 23707282

[ref112] Jurek B. Neumann I. D. (2018). The oxytocin receptor: from intracellular Signaling to behavior. Physiol. Rev. 98, 1805–1908. doi: 10.1152/physrev.00031.2017, 29897293

[ref113] Jurzak M. Müller A. R. Gerstberger R. (1995). Characterization of vasopressin receptors in cultured cells derived from the region of rat brain circumventricular organs. Neuroscience 65, 1145–1159. 7617168 10.1016/0306-4522(94)00539-h

[ref114] Kawada A. Nagasawa M. Murata A. Mogi K. Watanabe K. Kikusui T. (2019). Vasopressin enhances human preemptive strike in both males and females. Sci. Rep. 9:9664. doi: 10.1038/s41598-019-45953-y, 31273244 PMC6609689

[ref115] Kawasaki A. Hoshi K. Kawano M. Nogami H. Yoshikawa H. Hisano S. (2005). Up-regulation of VGLUT2 expression in hypothalamic-neurohypophysial neurons of the rat following osmotic challenge. Eur. J. Neurosci. 22, 672–680. doi: 10.1111/j.1460-9568.2005.04240.x, 16101749

[ref116] Kc P. Dick T. E. (2010). Modulation of cardiorespiratory function mediated by the paraventricular nucleus. Respir. Physiol. Neurobiol. 174, 55–64. doi: 10.1016/j.resp.2010.08.001, 20708107 PMC2967641

[ref117] Kc P. Haxhiu M. A. Tolentino-Silva F. P. Wu M. Trouth C. O. Mack S. O. (2002). Paraventricular vasopressin-containing neurons project to brain stem and spinal cord respiratory-related sites. Respir. Physiol. Neurobiol. 133, 75–88. doi: 10.1016/S1569-9048(02)00131-3, 12385733

[ref118] Kimi S. Maiti R. Srinivasan A. Mishra B. R. Hota D. (2024). Efficacy and safety of V_1a_ receptor antagonists in autism spectrum disorder: a meta-analysis. Int. J. Dev. Neurosci. 84, 3–13. doi: 10.1002/jdn.10297, 37641183

[ref119] King L. Robins S. Chen G. Yerko V. Zhou Y. Nagy C. (2017). Perinatal depression and DNA methylation of oxytocin-related genes: a study of mothers and their children. Horm. Behav. 96, 84–94. doi: 10.1016/j.yhbeh.2017.09.006, 28918249

[ref120] Knobloch H. S. Charlet A. Hoffmann L. C. Eliava M. Khrulev S. Cetin A. H. (2012). Evoked axonal oxytocin release in the central amygdala attenuates fear response. Neuron 73, 553–566. doi: 10.1016/j.neuron.2011.11.030, 22325206

[ref121] Koch S. B. Van Zuiden M. Nawijn L. Frijling J. L. Veltman D. J. Olff M. (2016). Intranasal oxytocin administration dampens amygdala reactivity towards emotional faces in male and female PTSD patients. Neuropsychopharmacol 41, 1495–1504. doi: 10.1038/npp.2015.299, 26404844 PMC4832009

[ref122] Koshimizu T. Nakamura K. Egashira N. Hiroyama M. Nonoguchi H. Tanoue A. (2012). Vasopressin V1a and V1b receptors: from molecules to physiological systems. Physiol. Rev. 92, 1813–1864. doi: 10.1152/physrev.00035.2011, 23073632

[ref123] Koshimizu T. Tsujimoto G. (2009). New topics in vasopressin receptors and approach to novel drugs: vasopressin and pain perception. J. Pharmacol. Sci. 109, 33–37. doi: 10.1254/jphs.08R18FM, 19151539

[ref124] Kou J. Zhang Y. Zhou F. Gao Z. Yao S. Zhao W. (2022). Anxiolytic effects of chronic intranasal oxytocin on neural responses to threat are dose-frequency dependent. Psychother. Psychosom. 91, 253–264. doi: 10.1159/000521348, 35086102

[ref125] Landgraf R. Neumann I. D. (2004). Vasopressin and oxytocin release within the brain: a dynamic concept of multiple and variable modes of neuropeptide communication. Front. Neuroendocrinol. 25, 150–176. doi: 10.1016/j.yfrne.2004.05.001, 15589267

[ref126] Le J. Zhang L. Zhao W. Zhu S. Lan C. Kou J. . (2022). Infrequent intranasal oxytocin followed by positive social interaction improves symptoms in autistic children: a pilot randomized clinical trial. Psychother. Psychosom. 91, 335–347. doi: 10.1159/000524543, 35545057

[ref127] Leng G. Ludwig M. (2016). Intranasal oxytocin: myths and delusions. Biol. Psychiatry 79, 243–250. doi: 10.1016/j.biopsych.2015.05.003, 26049207

[ref128] Leonzino M. Busnelli M. Antonucci F. Verderio C. Mazzanti M. Chini B. (2016). The timing of the excitatory-to-inhibitory GABA switch is regulated by the oxytocin receptor via KCC2. Cell Rep. 15, 96–103. doi: 10.1016/j.celrep.2016.03.013, 27052180 PMC4826440

[ref129] Leppanen J. Ng K. W. Tchanturia K. Treasure J. (2017). Meta-analysis of the effects of intranasal oxytocin on interpretation and expression of emotions. Neurosci. Biobehav. Rev. 78, 125–144. doi: 10.1016/j.neubiorev.2017.04.010, 28467893

[ref130] Leroy F. Brann D. H. Meira T. Siegelbaum S. A. (2019). Input-timing-dependent plasticity in the hippocampal CA2 region and its potential role in social memory. Neuron 102, 260–262. doi: 10.1016/j.neuron.2019.03.021, 30946821 PMC6563609

[ref131] Leroy F. Park J. Asok A. Brann D. H. Meira T. Boyle L. M. (2018). A circuit from hippocampal CA2 to lateral septum disinhibits social aggression. Nature 564, 213–218. doi: 10.1038/s41586-018-0772-0, 30518859 PMC6364572

[ref132] Lesse A. Rether K. Gröger N. Braun K. Bock J. (2017). Chronic postnatal stress induces depressive-like behavior in male mice and programs second-hit stress-induced gene expression patterns of OxtR and AvpR1a in adulthood. Mol. Neurobiol. 54, 4813–4819. doi: 10.1007/s12035-016-0043-8, 27525673

[ref133] Leung C. Cao F. Nguyen R. Joshi K. Aqrabawi A. J. Xia S. (2018). Activation of entorhinal cortical projections to the dentate gyrus underlies social memory retrieval. Cell Rep. 23, 2379–2391. doi: 10.1016/j.celrep.2018.04.073, 29791849

[ref134] Lin Y.-T. Hsieh T.-Y. Tsai T.-C. Chen C.-C. Huang C.-C. Hsu K.-S. (2018). Conditional deletion of hippocampal CA2/CA3a oxytocin receptors impairs the persistence of long-term social recognition memory in mice. J. Neurosci. 38, 1218–1231. doi: 10.1523/JNEUROSCI.1896-17.2017, 29279308 PMC6596267

[ref135] Liu C. M. Hsu T. M. Suarez A. N. Subramanian K. S. Fatemi R. A. Cortella A. M. (2020). Central oxytocin signaling inhibits food reward-motivated behaviors and VTA dopamine responses to food-predictive cues in male rats. Horm. Behav. 126:104855. doi: 10.1016/j.yhbeh.2020.104855, 32991888 PMC7757852

[ref136] Liu Y. Li S. Lin W. Li W. Yan X. Wang X. (2019). Oxytocin modulates social value representations in the amygdala. Nat. Neurosci. 22, 633–641. doi: 10.1038/s41593-019-0351-1, 30911182

[ref137] Liu X. Tribollet E. Ogier R. Barberis C. Raggenbass M. (2003). Presence of functional vasopressin receptors in spinal ventral horn neurons of young rats: a morphological and electrophysiological study. Eur. J. Neurosci. 17, 1833–1846. doi: 10.1046/j.1460-9568.2003.02625.x, 12752783

[ref138] Loewen S. P. Baimoukhametova D. V. Bains J. S. (2020). Sex-specific vasopressin Signaling buffers stress-dependent synaptic changes in female mice. J. Neurosci. 40, 8842–8852. doi: 10.1523/JNEUROSCI.1026-20.2020, 33051356 PMC7659452

[ref139] Lukas M. Bredewold R. Landgraf R. Neumann I. D. Veenema A. H. (2011). Early life stress impairs social recognition due to a blunted response of vasopressin release within the septum of adult male rats. Psychoneuroendocrinology 36, 843–853. doi: 10.1016/j.psyneuen.2010.11.007, 21185124

[ref140] Lukas M. Bredewold R. Neumann I. D. Veenema A. H. (2010). Maternal separation interferes with developmental changes in brain vasopressin and oxytocin receptor binding in male rats. Neuropharmacology 58, 78–87. doi: 10.1016/j.neuropharm.2009.06.020, 19560475

[ref141] Lukas M. Neumann I. D. (2014). Social preference and maternal defeat-induced social avoidance in virgin female rats: sex differences in involvement of brain oxytocin and vasopressin. J. Neurosci. Methods 234, 101–107. doi: 10.1016/j.jneumeth.2014.03.013, 24709115

[ref142] Lukas M. Toth I. Veenema A. H. Neumann I. D. (2013). Oxytocin mediates rodent social memory within the lateral septum and the medial amygdala depending on the relevance of the social stimulus: male juvenile versus female adult conspecifics. Psychoneuroendocrinology 38, 916–926. doi: 10.1016/j.psyneuen.2012.09.018, 23102690

[ref143] MacKinnon A. L. Gold I. Feeley N. Hayton B. Carter C. S. Zelkowitz P. (2014). The role of oxytocin in mothers’ theory of mind and interactive behavior during the perinatal period. Psychoneuroendocrinology 48, 52–63. doi: 10.1016/j.psyneuen.2014.06.003, 24995584

[ref144] MacLean E. L. Gesquiere L. R. Gee N. R. Levy K. Martin W. L. Carter C. S. (2017). Effects of affiliative human–animal interaction on dog salivary and plasma oxytocin and vasopressin. Front. Psychol. 8:1606. doi: 10.3389/fpsyg.2017.01606, 28979224 PMC5611686

[ref145] Marciniak A. Pacini L. Papini A. M. Brasuń J. (2022). Bicyclopeptides: a new class of ligands for cu(ii) ions. Dalton Trans. 51, 13368–13375. doi: 10.1039/D2DT01497A, 35984441

[ref146] Martínez-Lorenzana G. Palma-Tirado L. Cifuentes-Diaz C. González–Hernández, A Condés-Lara M. (2021). Ultrastructural evidence for oxytocin and oxytocin receptor at the spinal dorsal Horn: mechanism of nociception modulation. Neuroscience 475, 117–126. doi: 10.1016/j.neuroscience.2021.09.004, 34530103

[ref147] Martins D. Gabay A. S. Mehta M. Paloyelis Y. (2020). Salivary and plasmatic oxytocin are not reliable trait markers of the physiology of the oxytocin system in humans. eLife 9:e62456. doi: 10.7554/eLife.62456, 33306025 PMC7732341

[ref148] Matsui S. Takahashi Y. Morioka S. Ozawa T. Kanayama S. Iwama H. (2026). Negative feedback regulation of alcohol ingestion through the FGF21-PVH oxytocin-VTA dopamine system. Proc. Natl. Acad. Sci. USA 123:e2525172122. doi: 10.1073/pnas.2525172122, 41533444 PMC12818416

[ref149] Meinung C.-P. Boi L. Pandamooz S. Mazaud D. Ghézali G. Rouach N. (2025). OXTR-mediated signaling in astrocytes contributes to anxiolysis. Mol. Psychiatry 30, 2620–2634. doi: 10.1038/s41380-024-02870-5, 39702695 PMC12092269

[ref150] Meira T. Leroy F. Buss E. W. Oliva A. Park J. Siegelbaum S. A. (2018). A hippocampal circuit linking dorsal CA2 to ventral CA1 critical for social memory dynamics. Nat. Commun. 9:4163. doi: 10.1038/s41467-018-06501-w, 30301899 PMC6178349

[ref151] Menon R. Grund T. Zoicas I. Althammer F. Fiedler D. Biermeier V. . (2018). Oxytocin signaling in the lateral septum prevents social fear during lactation. Curr. Biol. 28, 1066–1078 e6. doi: 10.1016/j.cub.2018.02.044, 29551417

[ref152] Meziane H. Schaller F. Bauer S. Villard C. Matarazzo V. Riet F. (2015). An early postnatal oxytocin treatment prevents social and learning deficits in adult mice deficient for Magel2, a gene involved in Prader-Willi syndrome and autism. Biol. Psychiatry 78, 85–94. doi: 10.1016/j.biopsych.2014.11.010, 25599930

[ref153] Mittaud P. Labourdette G. Zingg H. Guenot-Di Scala D. (2002). Neurons modulate oxytocin receptor expression in rat cultured astrocytes: involvement of TGF-β and membrane components. Glia 37, 169–177. doi: 10.1002/glia.10029, 11754214

[ref154] Miyata S. Takamatsu H. Maekawa S. Matsumoto N. Watanabe K. Kiyohara T. (2001). Plasticity of neurohypophysial terminals with increased hormonal release during dehydration: ultrastructural and biochemical analyses. J. Comp. Neurol. 434, 413–427. doi: 10.1002/cne.1184, 11343290

[ref155] Mogil J. S. Sorge R. E. LaCroix-Fralish M. L. Smith S. B. Fortin A. Sotocinal S. G. (2011). Pain sensitivity and vasopressin analgesia are mediated by a gene-sex-environment interaction. Nat. Neurosci. 14, 1569–1573. doi: 10.1038/nn.2941, 22019732 PMC3225498

[ref156] Mohapatra A. N. Peles D. Netser S. Wagner S. (2024). Synchronized LFP rhythmicity in the social brain reflects the context of social encounters. Commun Biol 7:2. doi: 10.1038/s42003-023-05728-8, 38168971 PMC10761981

[ref157] Morisot N. Monier R. Le Moine C. Millan M. J. Contarino A. (2018). Corticotropin-releasing factor receptor 2-deficiency eliminates social behaviour deficits and vulnerability induced by cocaine. British J Pharmacol. 175, 1504–1518. doi: 10.1111/bph.14159, 29406581 PMC5900993

[ref001] Mühlethaler M. Charpak S. Dreifuss J. J. (1984). Contrasting effects of neurohypophysial peptides on pyramidal and non-pyramidal neurones in the rat hippocampus. Brain Res. 308, 97–107. doi: 10.1016/0006-8993(84)90921-1, 6478205

[ref158] Musardo S. Contestabile A. Knoop M. Baud O. Bellone C. (2022). Oxytocin neurons mediate the effect of social isolation via the VTA circuits. eLife 11:e73421. doi: 10.7554/eLife.73421, 35451958 PMC9075949

[ref159] Nair H. P. Gutman A. R. Davis M. Young L. J. (2005). Central oxytocin, vasopressin, and corticotropin-releasing factor receptor densities in the basal forebrain predict isolation potentiated startle in rats. J. Neurosci. 25, 11479–11488. doi: 10.1523/JNEUROSCI.2524-05.2005, 16339041 PMC6725901

[ref160] Nakamura K. Karasawa K. Yasui M. Nuriya M. (2022). Probing the spatiotemporal dynamics of oxytocin in the brain tissue using a simple peptide alkyne-tagging approach. Anal. Chem. 94, 11990–11998. doi: 10.1021/acs.analchem.2c00452, 36008880

[ref161] Nave G. Camerer C. McCullough M. (2015). Does oxytocin increase Trust in Humans? A critical review of research. Perspect. Psychol. Sci. 10, 772–789. doi: 10.1177/1745691615600138, 26581735

[ref162] Nazarloo H. P. Kingsbury M. A. Lamont H. Dale C. V. Nazarloo P. Davis J. M. (2025). Oxytocin, vasopressin and stress: a Hormetic perspective. CIMB 47:632. doi: 10.3390/cimb47080632, 40864786 PMC12384123

[ref163] Neumann I. D. Landgraf R. (2012). Balance of brain oxytocin and vasopressin: implications for anxiety, depression, and social behaviors. Trends Neurosci. 35, 649–659. doi: 10.1016/j.tins.2012.08.004, 22974560

[ref164] Niermann H. Amiry-Moghaddam M. Holthoff K. Witte O. W. Ottersen O. P. (2001). A novel role of vasopressin in the brain: modulation of activity-dependent water flux in the neocortex. J. Neurosci. 21, 3045–3051. doi: 10.1523/JNEUROSCI.21-09-03045.2001, 11312289 PMC6762582

[ref165] Oettl L.-L. Ravi N. Schneider M. Scheller M. F. Schneider P. Mitre M. (2016). Oxytocin enhances social recognition by modulating cortical control of early olfactory processing. Neuron 90, 609–621. doi: 10.1016/j.neuron.2016.03.033, 27112498 PMC4860033

[ref166] Oliet S. H. R. Piet R. Poulain D. A. Theodosis D. T. (2004). Glial modulation of synaptic transmission: insights from the supraoptic nucleus of the hypothalamus. Glia 47, 258–267. doi: 10.1002/glia.20032, 15252815

[ref167] Oliveira V. E. M. Lukas M. Wolf H. N. Durante E. Lorenz A. Mayer A. L. . (2021). Oxytocin and vasopressin within the ventral and dorsal lateral septum modulate aggression in female rats. Nat. Commun. 12:2900. doi: 10.1038/s41467-021-23064-5, 34006875 PMC8131389

[ref168] Oliveira V. E. D. M. Neumann I. D. De Jong T. R. (2019). Post-weaning social isolation exacerbates aggression in both sexes and affects the vasopressin and oxytocin system in a sex-specific manner. Neuropharmacology 156:107504. doi: 10.1016/j.neuropharm.2019.01.019, 30664846

[ref169] Osakada T. Yan R. Jiang Y. Wei D. Tabuchi R. Dai B. (2024). A dedicated hypothalamic oxytocin circuit controls aversive social learning. Nature 626, 347–356. doi: 10.1038/s41586-023-06958-w, 38267576 PMC11102773

[ref170] Osako Y. Otsuka T. Taniguchi M. Oka T. Kaba H. (2001). Oxytocin enhances presynaptic and postsynaptic glutamatergic transmission between rat olfactory bulb neurones in culture. Neurosci. Lett. 299, 65–68. doi: 10.1016/S0304-3940(00)01779-1, 11166939

[ref171] Otubo A. Maejima S. Oti T. Satoh K. Ueda Y. Morris J. F. (2021). Immunoelectron microscopic characterization of vasopressin-producing neurons in the Hypothalamo-pituitary Axis of non-human Primates by use of formaldehyde-fixed tissues stored at −25 °C for several years. IJMS 22:9180. doi: 10.3390/ijms22179180, 34502087 PMC8430530

[ref172] Owen S. F. Tuncdemir S. N. Bader P. L. Tirko N. N. Fishell G. Tsien R. W. (2013). Oxytocin enhances hippocampal spike transmission by modulating fast-spiking interneurons. Nature 500, 458–462. doi: 10.1038/nature12330, 23913275 PMC5283693

[ref173] Oztan O. Garner J. P. Constantino J. N. Parker K. J. (2020). Neonatal CSF vasopressin concentration predicts later medical record diagnoses of autism spectrum disorder. Proc. Natl. Acad. Sci. USA 117, 10609–10613. doi: 10.1073/pnas.1919050117, 32341146 PMC7229671

[ref174] Pagani J. H. Zhao M. Cui Z. Williams Avram S. K. Caruana D. A. Dudek S. M. (2015). Role of the vasopressin 1b receptor in rodent aggressive behavior and synaptic plasticity in hippocampal area CA2. Mol. Psychiatry 20, 490–499. doi: 10.1038/mp.2014.47, 24863146 PMC4562468

[ref175] Pantouli F. Pujol C. N. Derieux C. Fonteneau M. Pellissier L. P. Marsol C. (2024). Acute, chronic and conditioned effects of intranasal oxytocin in the mu-opioid receptor knockout mouse model of autism: social context matters. Neuropsychopharmacol. 49, 1934–1946. doi: 10.1038/s41386-024-01915-1, 39020142 PMC11473707

[ref176] Parker K. J. Oztan O. Libove R. A. Mohsin N. Karhson D. S. Sumiyoshi R. D. (2019). A randomized placebo-controlled pilot trial shows that intranasal vasopressin improves social deficits in children with autism. Sci. Transl. Med. 11:eaau7356. doi: 10.1126/scitranslmed.aau7356, 31043522 PMC6716148

[ref177] Penagarikano O. Lazaro M. T. Lu X. H. Gordon A. Dong H. Lam H. A. . (2015). Exogenous and evoked oxytocin restores social behavior in the Cntnap2 mouse model of autism. Sci. Transl. Med. 7:271ra8. doi: 10.1126/scitranslmed.3010257, 25609168 PMC4498455

[ref178] Peng F. Qu Z.-W. Qiu C.-Y. Liao M. Hu W.-P. (2015). Spinal vasopressin alleviates formalin-induced nociception by enhancing GABAA receptor function in mice. Neurosci. Lett. 593, 61–65. doi: 10.1016/j.neulet.2015.03.023, 25782631

[ref179] Petersson M. Höybye C. (2024). Is oxytocin a contributor to Behavioral and metabolic features in Prader-Willi syndrome? CIMB 46, 8767–8779. doi: 10.3390/cimb46080518, 39194735 PMC11353121

[ref180] Petitjean H. Finck S. Schmoll P. Charlet A. (2025). The concept of homeodynamics in systems theory. Complexities 1:6. doi: 10.3390/complexities1010006

[ref181] Piet R. Vargová L. Syková E. Poulain D. A. Oliet S. H. R. (2004). Physiological contribution of the astrocytic environment of neurons to intersynaptic crosstalk. Proc. Natl. Acad. Sci. USA 101, 2151–2155. doi: 10.1073/pnas.0308408100, 14766975 PMC357067

[ref182] Piotrowska D. Potasiewicz A. Popik P. Nikiforuk A. (2024). Pro-social and pro-cognitive effects of LIT-001, a novel oxytocin receptor agonist in a neurodevelopmental model of schizophrenia. Eur. Neuropsychopharmacol. 78, 30–42. doi: 10.1016/j.euroneuro.2023.09.005, 37866191

[ref183] Pisansky M. T. Hanson L. R. Gottesman I. I. Gewirtz J. C. (2017). Oxytocin enhances observational fear in mice. Nat. Commun. 8:2102. doi: 10.1038/s41467-017-02279-5, 29235461 PMC5727393

[ref184] Raam T. McAvoy K. M. Besnard A. Veenema A. H. Sahay A. (2017). Hippocampal oxytocin receptors are necessary for discrimination of social stimuli. Nat. Commun. 8:2001. doi: 10.1038/s41467-017-02173-0, 29222469 PMC5722862

[ref185] Raggenbass M. (2008). Overview of cellular electrophysiological actions of vasopressin. Eur. J. Pharmacol. 583, 243–254. doi: 10.1016/j.ejphar.2007.11.074, 18280467

[ref186] Rastogi K. Weerts E. M. Ellis J. D. (2024). Oxytocin as a treatment for alcohol use disorder and heavy drinking: a narrative review. Exp. Clin. Psychopharmacol. 32, 625–638. doi: 10.1037/pha0000741, 39298263 PMC11995404

[ref187] Reijnen A. Geuze E. Vermetten E. (2017). Individual variation in plasma oxytocin and vasopressin levels in relation to the development of combat-related PTSD in a large military cohort. J. Psychiatr. Res. 94, 88–95. doi: 10.1016/j.jpsychires.2017.06.010, 28689067

[ref188] Rice L. J. Einfeld S. L. Hu N. Carter C. S. (2018). A review of clinical trials of oxytocin in Prader-Willi syndrome. Curr. Opin. Psychiatry 31, 123–127. doi: 10.1097/YCO.0000000000000391, 29206687

[ref189] Rigney N. Campos-Lira E. Kirchner M. K. Wei W. Belkasim S. Beaumont R. (2024). A vasopressin circuit that modulates mouse social investigation and anxiety-like behavior in a sex-specific manner. Proc. Natl. Acad. Sci. USA 121:e2319641121. doi: 10.1073/pnas.2319641121, 38709918 PMC11098102

[ref190] Rigney N. Whylings J. Mieda M. de Vries G. Petrulis A. (2019). Sexually dimorphic vasopressin cells modulate social investigation and communication in sex-specific ways. eNeuro 6:e0415–18.2019. doi: 10.1523/ENEURO.0415-18.2019, 30693316 PMC6348451

[ref191] Rizzi-Wise C. A. Wang D. V. (2021). Putting together pieces of the lateral septum: multifaceted functions and its neural pathways. eNeuro 8:ENEURO.0315-21.2021. doi: 10.1523/ENEURO.0315-21.2021, 34764187 PMC8647703

[ref192] Rossoni E. Feng J. Tirozzi B. Brown D. Leng G. Moos F. (2008). Emergent synchronous bursting of oxytocin neuronal network. PLoS Comput. Biol. 4:e1000123. doi: 10.1371/journal.pcbi.1000123, 18636098 PMC2440533

[ref193] Rutigliano G. Rocchetti M. Paloyelis Y. Gilleen J. Sardella A. Cappucciati M. (2016). Peripheral oxytocin and vasopressin: biomarkers of psychiatric disorders? A comprehensive systematic review and preliminary meta-analysis. Psychiatry Res. 241, 207–220. doi: 10.1016/j.psychres.2016.04.117, 27183106

[ref194] Ryan P. J. Ross S. I. Campos C. A. Derkach V. A. Palmiter R. D. (2017). Oxytocin-receptor-expressing neurons in the parabrachial nucleus regulate fluid intake. Nat. Neurosci. 20, 1722–1733. doi: 10.1038/s41593-017-0014-z, 29184212 PMC5705772

[ref195] Sack M. Spieler D. Wizelman L. Epple G. Stich J. Zaba M. (2017). Intranasal oxytocin reduces provoked symptoms in female patients with posttraumatic stress disorder despite exerting sympathomimetic and positive chronotropic effects in a randomized controlled trial. BMC Med. 15:40. doi: 10.1186/s12916-017-0801-0, 28209155 PMC5314583

[ref196] Sala M. Braida D. Lentini D. Busnelli M. Bulgheroni E. Capurro V. (2011). Pharmacologic rescue of impaired cognitive flexibility, social deficits, increased aggression, and seizure susceptibility in oxytocin receptor null mice: a neurobehavioral model of autism. Biol. Psychiatry 69, 875–882. doi: 10.1016/j.biopsych.2010.12.022, 21306704

[ref197] Salles J. Eddiry S. Amri S. Galindo M. Lacassagne E. George S. (2024). Differential DNA methylation in iPSC-derived dopaminergic neurons: a step forward on the role of SNORD116 microdeletion in the pathophysiology of addictive behavior in Prader-Willi syndrome. Mol. Psychiatry 29, 2742–2752. doi: 10.1038/s41380-024-02542-4, 38561465

[ref198] Sandoval K. C. Rychlik J. Choe K. Y. (2025). Calcium dynamics in hypothalamic paraventricular oxytocin neurons and astrocytes associated with social and stress stimuli. eNeuro 12:ENEURO.0196-24.2025. doi: 10.1523/ENEURO.0196-24.2025, 40262904 PMC12071343

[ref199] Sawchenko P. E. Swanson L. W. (1982). Immunohistochemical identification of neurons in the paraventricular nucleus of the hypothalamus that project to the medulla or to the spinal cord in the rat. J. Comp. Neurol. 205, 260–272. 6122696 10.1002/cne.902050306

[ref200] Schmidt M. Braun K. Brandwein C. Rossetti A. C. Guara Ciurana S. Riva M. A. (2018). Maternal stress during pregnancy induces depressive-like behavior only in female offspring and correlates to their hippocampal Avp and Oxt receptor expression. Behav. Brain Res. 353, 1–10. doi: 10.1016/j.bbr.2018.06.027, 29958961

[ref201] Schöneberg T. Kostenis E. Liu J. Gudermann T. Wess J. (1998). Molecular aspects of vasopressin receptor function. Adv. Exp. Med. Biol. 449, 347–358. 10026824 10.1007/978-1-4615-4871-3_44

[ref202] Schorscher-Petcu A. Sotocinal S. Ciura S. Dupré A. Ritchie J. Sorge R. E. . (2010). Oxytocin-induced analgesia and scratching are mediated by the vasopressin-1A receptor in the mouse. J. Neurosci. 30, 8274–8284. doi: 10.1523/JNEUROSCI.1594-10.2010, 20554879 PMC2902996

[ref203] Shalma N. M. Alsharabasy M. A. Taha A. M. Alsawareah A. Manirambona E. Ahmed S. K. (2023). The efficacy of intranasal oxytocin in patients with Prader-Willi syndrome: a systematic review and meta-analysis. Diabetes Metab. Syndr. Clin. Res. Rev. 17:102711. doi: 10.1016/j.dsx.2023.102711, 36774885

[ref204] Sikich L. Kolevzon A. King B. H. McDougle C. J. Sanders K. B. Kim S.-J. (2021). Intranasal oxytocin in children and adolescents with autism Spectrum disorder. N. Engl. J. Med. 385, 1462–1473. doi: 10.1056/NEJMoa2103583, 34644471 PMC9701092

[ref205] Sippel L. M. Wachsman T. R. Kelley M. E. Knopp K. C. Khalifian C. E. Maglione J. E. (2024). Design of a randomized clinical trial of brief couple therapy for PTSD augmented with intranasal oxytocin. Contemp. Clin. Trials 141:107534. doi: 10.1016/j.cct.2024.107534, 38614447

[ref206] Skuse D. H. Gallagher L. (2009). Dopaminergic-neuropeptide interactions in the social brain. Trends Cogn. Sci. 13, 27–35. doi: 10.1016/j.tics.2008.09.007, 19084465

[ref207] Smith C. J. W. DiBenedictis B. T. Veenema A. H. (2019). Comparing vasopressin and oxytocin fiber and receptor density patterns in the social behavior neural network: implications for cross-system signaling. Front. Neuroendocrinol. 53:100737. doi: 10.1016/j.yfrne.2019.02.001, 30753840 PMC7469073

[ref208] Smith C. J. W. Poehlmann M. L. Li S. Ratnaseelan A. M. Bredewold R. Veenema A. H. (2017). Age and sex differences in oxytocin and vasopressin V1a receptor binding densities in the rat brain: focus on the social decision-making network. Brain Struct. Funct. 222, 981–1006. doi: 10.1007/s00429-016-1260-7, 27389643 PMC5334374

[ref209] Song Z. Albers H. E. (2018). Cross-talk among oxytocin and arginine-vasopressin receptors: relevance for basic and clinical studies of the brain and periphery. Front. Neuroendocrinol. 51, 14–24. doi: 10.1016/j.yfrne.2017.10.004, 29054552 PMC5906207

[ref210] Song Z. Borland J. M. Larkin T. E. O’Malley M. Albers H. E. (2016). Activation of oxytocin receptors, but not arginine-vasopressin V1a receptors, in the ventral tegmental area of male Syrian hamsters is essential for the reward-like properties of social interactions. Psychoneuroendocrinology 74, 164–172. doi: 10.1016/j.psyneuen.2016.09.001, 27632574 PMC6417503

[ref211] Song S. C. Froemke R. C. (2025). Lateralized local circuit tuning in female mouse auditory cortex. Neurosci. Res. 216:104897. doi: 10.1016/j.neures.2025.03.009, 40189152 PMC12174909

[ref212] Spengler F. B. Schultz J. Scheele D. Essel M. Maier W. Heinrichs M. (2017). Kinetics and dose dependency of intranasal oxytocin effects on amygdala reactivity. Biol. Psychiatry 82, 885–894. doi: 10.1016/j.biopsych.2017.04.015, 28629540

[ref213] Stoop R. (2012). Neuromodulation by oxytocin and vasopressin. Neuron 76, 142–159. doi: 10.1016/j.neuron.2012.09.025, 23040812

[ref214] Stoop R. Hegobuu C. van der Burg E. (2015). New opportunities in vasopressin and oxytocin research: a perspective from the amygdala. Annu. Rev. Neurosci. 38, 369–388. doi: 10.1146/annurev-neuro-071714-033904, 26154981

[ref215] Sun Y. Qian W. Zhang L. Niu H. Li L. Li L. (2025). Tactile stimulation reverses painful stimuli outcomes via PVN-VTA oxytocin circuitry and dopaminergic regulation. Commun Biol 8:1799. doi: 10.1038/s42003-025-09208-z, 41310147 PMC12722742

[ref216] Surget A. Belzung C. (2008). Involvement of vasopressin in affective disorders. Eur. J. Pharmacol. 583, 340–349. doi: 10.1016/j.ejphar.2007.11.065, 18255056

[ref217] Tabak B. A. Leng G. Szeto A. Parker K. J. Verbalis J. G. Ziegler T. E. (2023). Advances in human oxytocin measurement: challenges and proposed solutions. Mol. Psychiatry 28, 127–140. doi: 10.1038/s41380-022-01719-z, 35999276 PMC9812775

[ref218] Talbot C. F. Oztan O. Simmons S. M. V. Trainor C. Ceniceros L. C. Nguyen D. K. K. (2024). Nebulized vasopressin penetrates CSF and improves social cognition without inducing aggression in a rhesus monkey model of autism. Proc. Natl. Acad. Sci. USA 121:e2418635121. doi: 10.1073/pnas.2418635121, 39585977 PMC11626171

[ref219] Tang Y. Benusiglio D. Lefevre A. Hilfiger L. Althammer F. Bludau A. (2020). Social touch promotes interfemale communication via activation of parvocellular oxytocin neurons. Nat. Neurosci. 23, 1125–1137. doi: 10.1038/s41593-020-0674-y, 32719563

[ref220] Tasker J. G. Oliet S. H. R. Bains J. S. Brown C. H. Stern J. E. (2012). Glial regulation of neuronal function: from synapse to systems physiology. J. Neuroendocrinol. 24, 566–576. doi: 10.1111/j.1365-2826.2011.02259.x, 22128866 PMC3314084

[ref221] Tauber M. Boulanouar K. Diene G. Cabal-Berthoumieu S. Ehlinger V. Fichaux-Bourin P. . (2017). The use of oxytocin to improve feeding and social skills in infants with Prader-Willi syndrome. Pediatrics 139:e20162976. doi: 10.1542/peds.2016-2976, 28100688

[ref222] Teixeira G. P. Rocha L. Faria R. X. (2025). The impact of membrane receptors on modulating empathic pain. Neuropharmacology 274:110471. doi: 10.1016/j.neuropharm.2025.110471, 40254122

[ref223] Tendler A. Wagner S. (2015). Different types of theta rhythmicity are induced by social and fearful stimuli in a network associated with social memory. eLife 4, 1–22. doi: 10.7554/eLife.03614, 25686218 PMC4353977

[ref224] Terrill S. J. Holt M. K. Maske C. B. Abrams N. Reimann F. Trapp S. (2019). Endogenous GLP-1 in lateral septum promotes satiety and suppresses motivation for food in mice. Physiol. Behav. 206, 191–199. doi: 10.1016/j.physbeh.2019.04.008, 30980855 PMC6956655

[ref225] Terrill S. J. Maske C. B. Williams D. L. (2018). Endogenous GLP-1 in lateral septum contributes to stress-induced hypophagia. Physiol. Behav. 192, 17–22. doi: 10.1016/j.physbeh.2018.03.001, 29510158 PMC6019151

[ref226] Thirtamara Rajamani K. Barbier M. Lefevre A. Niblo K. Cordero N. Netser S. (2024). Oxytocin activity in the paraventricular and supramammillary nuclei of the hypothalamus is essential for social recognition memory in rats. Mol. Psychiatry 29, 412–424. doi: 10.1038/s41380-023-02336-0, 38052983 PMC11116117

[ref227] Tirko N. N. Eyring K. W. Carcea I. Mitre M. Chao M. V. Froemke R. C. . (2018). Oxytocin transforms firing mode of CA2 hippocampal neurons. Neuron 100, 593–608 e3. doi: 10.1016/j.neuron.2018.09.008, 30293821 PMC6516476

[ref228] Tobin V. A. Hashimoto H. Wacker D. W. Takayanagi Y. Langnaese K. Caquineau C. (2010). An intrinsic vasopressin system in the olfactory bulb is involved in social recognition. Nature 464, 413–417. doi: 10.1038/nature08826, 20182426 PMC2842245

[ref229] Tribollet E. Charpak S. Schmidt A. Dubois-Dauphin M. Dreifuss J. (1989). Appearance and transient expression of oxytocin receptors in fetal, infant, and peripubertal rat brain studied by autoradiography and electrophysiology. J. Neurosci. 9, 1764–1773. 2542479 10.1523/JNEUROSCI.09-05-01764.1989 PMC6569831

[ref230] Urban I. J. (1981). Intraseptal administration of vasopressin and oxytocin affects hippocampal electroencephalogram in rats. Exp. Neurol. 74, 131–147. 7286113 10.1016/0014-4886(81)90154-0

[ref231] Urban I. J. (1998). Effects of vasopressin and related peptides on neurons of the rat lateral septum and ventral hippocampus. Prog. Brain Res. 119, 285–310. 10074795 10.1016/s0079-6123(08)61576-9

[ref232] Urban I. J. De Wied D. (1986). Effect of vasopressin, oxytocin and peptides derived from these hormones on field potential induced in lateral septum of rats by stimulation of the fimbria fornix. Neuropeptides 7, 41–49. 3951680 10.1016/0143-4179(86)90078-8

[ref233] Uzefovsky F. Shalev I. Israel S. Edelman S. Raz Y. Mankuta D. (2015). Oxytocin receptor and vasopressin receptor 1a genes are respectively associated with emotional and cognitive empathy. Horm. Behav. 67, 60–65. doi: 10.1016/j.yhbeh.2014.11.007, 25476609

[ref234] Valette M. Diene G. Glattard M. Cortadellas J. Molinas C. Faye S. . (2025). Early oxytocin treatment in infants with Prader–Willi syndrome is safe and is associated with better endocrine, metabolic and behavioral outcomes. Orphanet J. Rare Dis. 20, 2–11. doi: 10.1186/s13023-025-03560-3, 40025514 PMC11872305

[ref235] Van den Hooff P. Urban I. J. (1990). Vasopressin facilitates excitatory transmission in slices of the rat dorso-lateral septum. Synapse 5, 201–206. 1971460 10.1002/syn.890050305

[ref236] van den Pol A. N. (2012). Neuropeptide transmission in brain circuits. Neuron 76, 98–115. doi: 10.1016/j.neuron.2012.09.014, 23040809 PMC3918222

[ref237] Van Wimersma Greidanus T. B. Maigret C. (1996). The role of limbic vasopressin and oxytocin in social recognition. Brain Res. 713, 153–159. 8724986 10.1016/0006-8993(95)01505-1

[ref238] Van Zuiden M. Frijling J. L. Nawijn L. Koch S. B. J. Goslings J. C. Luitse J. S. . (2017). Intranasal oxytocin to prevent posttraumatic stress disorder symptoms: a randomized controlled trial in emergency department patients. Biol. Psychiatry 81, 1030–1040. doi: 10.1016/j.biopsych.2016.11.012, 28087128

[ref239] Veening J. G. Olivier B. (2013). Intranasal administration of oxytocin: Behavioral and clinical effects, a review. Neurosci. Biobehav. Rev. 37, 1445–1465. doi: 10.1016/j.neubiorev.2013.04.012, 23648680 PMC7112651

[ref240] Viaux-Savelon S. Rosenblum O. Guedeney A. Diene G. Çabal-Berthoumieu S. Fichaux-Bourin P. (2016). Dyssynchrony and perinatal psychopathology impact of child disease on parents-child interactions, the paradigm of Prader Willi syndrom. J. Physiol.-Paris 110, 427–433. doi: 10.1016/j.jphysparis.2017.08.001, 28823614

[ref241] Viviani D. Charlet A. Van Den Burg E. Robinet C. Hurni N. Abatis M. . (2011). Oxytocin selectively gates fear responses through distinct outputs from the central amygdala. Science 333, 104–107. doi: 10.1126/science.1201043, 21719680

[ref242] Wacker D. W. Ludwig M. (2012). Vasopressin, oxytocin, and social odor recognition. Horm. Behav. 61, 259–265. doi: 10.1016/j.yhbeh.2011.08.014, 21920364

[ref243] Wahis J. Baudon A. Althammer F. Kerspern D. Goyon S. Hagiwara D. (2021). Astrocytes mediate the effect of oxytocin in the central amygdala on neuronal activity and affective states in rodents. Nat. Neurosci. 24, 529–541. doi: 10.1038/s41593-021-00800-0, 33589833

[ref244] Wang S.-C. Lin C.-C. Chen C.-C. Liu Y.-P. (2025). Acute restraint stress enhances prosocial behavior in rats via oxytocin and fear-related circuits. J. Integr. Neurosci. 24:33400. doi: 10.31083/JIN33400, 40302260

[ref245] Wang S.-C. Lin C.-C. Tzeng N.-S. Tung C.-S. Liu Y.-P. (2019). Effects of oxytocin on prosocial behavior and the associated profiles of oxytocinergic and corticotropin-releasing hormone receptors in a rodent model of posttraumatic stress disorder. J. Biomed. Sci. 26:26. doi: 10.1186/s12929-019-0514-0, 30898126 PMC6427848

[ref246] Welker P. Böhlick A. Mutig K. Salanova M. Kahl T. Schlüter H. (2008). Renal Na^+^ -K^+^ -cl^−^ cotransporter activity and vasopressin-induced trafficking are lipid raft-dependent. American J. Physiology-Renal Physiol. 295, F789–F802. doi: 10.1152/ajprenal.90227.2008, 18579701 PMC2536870

[ref247] Whylings J. Rigney N. De Vries G. J. Petrulis A. (2021). Removal of vasopressin cells from the paraventricular nucleus of the hypothalamus enhances lipopolysaccharide-induced sickness behaviour in mice. J. Neuroendocrinol. 33:e12915. doi: 10.1111/jne.12915, 33617060 PMC8543850

[ref248] Winter J. Jurek B. (2019). The interplay between oxytocin and the CRF system: regulation of the stress response. Cell Tissue Res. 375, 85–91. doi: 10.1007/s00441-018-2866-2, 29911261

[ref249] Winter J. Meyer M. Berger I. Royer M. Bianchi M. Kuffner K. (2023). Chronic oxytocin-driven alternative splicing of Crfr2α induces anxiety. Mol. Psychiatry 28, 4742–4755. doi: 10.1038/s41380-021-01141-x, 34035479 PMC10914602

[ref250] Wong L. C. Wang L. D’Amour J. A. Yumita T. Chen G. Yamaguchi T. (2016). Effective modulation of male aggression through lateral septum to medial hypothalamus projection. Curr. Biol. 26, 593–604. doi: 10.1016/j.cub.2015.12.065, 26877081 PMC4783202

[ref251] Wrobel L. J. Reymond-Marron I. Dupré A. Raggenbass M. (2010). Oxytocin and vasopressin enhance synaptic transmission in the hypoglossal motor nucleus of young rats by acting on distinct receptor types. Neuroscience 165, 723–735. doi: 10.1016/j.neuroscience.2009.11.001, 19896520

[ref252] Xiao L. Priest M. F. Nasenbeny J. Lu T. Kozorovitskiy Y. (2017). Biased oxytocinergic modulation of midbrain dopamine systems. Neuron 95, 368–384.e5. doi: 10.1016/j.neuron.2017.06.003, 28669546 PMC7881764

[ref253] Yang X. Wang W. Wang X. T. Wang Y. W. (2021). A meta-analysis of hormone administration effects on cooperative behaviours: oxytocin, vasopressin, and testosterone. Neurosci. Biobehav. Rev. 126, 430–443. doi: 10.1016/j.neubiorev.2021.03.033, 33819546

[ref254] Zanos P. Georgiou P. Wright S. R. Hourani S. M. Kitchen I. (2014). The oxytocin analogue Carbetocin prevents emotional impairment and stress-induced reinstatement of opioid-seeking in morphine-abstinent mice. Neuropsychopharmacol 39, 855–865. doi: 10.1038/npp.2013.285, 24129263 PMC3924520

[ref255] Zhang L. Hernández V. S. Zetter M. A. Eiden L. E. (2020). VGLUT-VGAT expression delineates functionally specialised populations of vasopressin-containing neurones including a glutamatergic perforant path-projecting cell group to the hippocampus in rat and mouse brain. J. Neuroendocrinol. 32:e12831. doi: 10.1111/jne.12831, 31944441 PMC13088980

[ref256] Zhang Y. Karadas M. Liu J. Gu X. Vöröslakos M. Li Y. (2024). Interaction of acetylcholine and oxytocin neuromodulation in the hippocampus. Neuron 112, 1862–1875.e5. doi: 10.1016/j.neuron.2024.02.021, 38537642 PMC11156550

[ref257] Zhao D.-Q. Ai H.-B. (2011). Oxytocin and vasopressin involved in restraint water-immersion stress mediated by oxytocin receptor and vasopressin 1b receptor in rat brain. PLoS One 6:e23362. doi: 10.1371/journal.pone.0023362, 21858088 PMC3157380

[ref258] Zhao L. Brinton R. D. (2003). Vasopressin-induced cytoplasmic and nuclear calcium Signaling in embryonic cortical astrocytes: dynamics of calcium and calcium-dependent kinase translocation. J. Neurosci. 23, 4228–4239. doi: 10.1523/JNEUROSCI.23-10-04228.2003, 12764111 PMC6741105

[ref259] Zhao W. Zhang X. Kendrick K. M. (2026). From fundamental research to clinical translation: the neural modulation of social behavior by oxytocin and vasopressin. Neurosci. Biobehav. Rev. 185:106624. doi: 10.1016/j.neubiorev.2026.106624, 41796878

[ref260] Zheng H. Lim J. Y. Kim Y. Jung S. T. Hwang S. W. (2021). The role of oxytocin, vasopressin, and their receptors at nociceptors in peripheral pain modulation. Front. Neuroendocrinol. 63:100942. doi: 10.1016/j.yfrne.2021.100942, 34437871

[ref261] Zhuang Q. Zheng X. Becker B. Lei W. Xu X. Kendrick K. M. (2021). Intranasal vasopressin like oxytocin increases social attention by influencing top-down control, but additionally enhances bottom-up control. Psychoneuroendocrinology 133:105412. doi: 10.1016/j.psyneuen.2021.105412, 34537624

[ref262] Zink C. F. Meyer-Lindenberg A. (2012). Human neuroimaging of oxytocin and vasopressin in social cognition. Horm. Behav. 61, 400–409. doi: 10.1016/j.yhbeh.2012.01.016, 22326707 PMC3312952

[ref263] Zink C. F. Stein J. L. Kempf L. Hakimi S. Meyer-Lindenberg A. (2010). Vasopressin modulates medial prefrontal cortex-amygdala circuitry during emotion processing in humans. J. Neurosci. 30, 7017–7022. doi: 10.1523/JNEUROSCI.4899-09.2010, 20484643 PMC2880169

[ref264] Zylbertal A. Yarom Y. Wagner S. (2017). Synchronous infra-slow bursting in the mouse accessory olfactory bulb emerge from interplay between intrinsic neuronal dynamics and network connectivity. J. Neurosci. 37, 2656–2672. doi: 10.1523/JNEUROSCI.3107-16.2017, 28148726 PMC6596634

